# A reusable model of pangenome selection informs optimal surveillance strategies over vaccine introductions

**DOI:** 10.1186/s13073-026-01672-4

**Published:** 2026-09-07

**Authors:** Leonie J. Lorenz, Joel Hellewell, Samuel T. Horsfield, Matthew J. Russell, Shrijana Shrestha, Andrew J. Pollard, Stephen D. Bentley, Stephanie W. Lo, Caroline Colijn, Nicholas J. Croucher, John A. Lees

**Affiliations:** 1 European Bioinformatics Institute, European Molecular Biology Laboratory, Wellcome Genome Campus, CB10 1SD Hinxton, Cambridge UK; 2 School of Engineering Mathematics and Technology, BS8 1TW Bristol, UK; 3 Institute of Biology, University of Neuchâtel, Rue Émile-Argand 11, Neuchâtel, 2000 Switzerland; 4 Patan Academy of Health Sciences, Lalitpur, Nepal; 5 Oxford Vaccine Group, Department of Paediatrics, University of Oxford, Oxford, UK; 6 The Wellcome Trust Sanger Institute, Wellcome Trust Genome Campus, CB10 1SA Hinxton, Cambridge UK; 7 Department of Medicine, University of Cambridge, Addenbrooke’s Hospital, CB2 0QQ Cambridge, UK; 8 Department of Mathematics, Simon Fraser University, V5A 1S6 Burnaby, BC Canada; 9 MRC Centre for Global Infectious Disease Analysis, Department of Infectious Disease Epidemiology, Imperial College London, W12 0BZ London, UK

**Keywords:** *Streptococcus pneumoniae*, Negative frequency-dependent selection, NFDS, Wright-Fisher model, Mathematical modelling, Vaccine replacement

## Abstract

**Background:**

The human pathogen *Streptococcus pneumoniae* is a major cause of disease, including pneumonia and meningitis. The introduction of Pneumococcal Conjugate Vaccines (PCVs) initially reduced the burden of disease through a reduction of colonisation by vaccine-targeted serotypes. However, since PCVs only target a proportion of pneumococcal serotypes, they shift intraspecific competition, eventually allowing non-targeted types to ’replace’ vaccine types. Understanding the host and pathogen factors causing replacement is important for future vaccine development. Mechanistic understanding of vaccine replacement dynamics is crucial for forecasting and optimisation of genomic surveillance strategies to evaluate realised vaccine effectiveness.

**Methods:**

We developed a mathematical model of the genomic and demographic factors which explain vaccine replacement, used this model to replicate serotype-frequency changes, and investigated cost-effective genomic surveillance strategies. We extended a forward-time model based on the Wright-Fisher model, developing a user-friendly model framework that describes the post-vaccine dynamics of *S. pneumoniae* populations. Our model describes vaccine replacement as a function of vaccine impact, immigration of new strains, and negative frequency-dependent selection (NFDS) on the accessory genome content.

**Results:**

We used our model to study vaccine replacement in newly sequenced genomic surveillance data from Kathmandu (Nepal), and existing data from Massachusetts (US) and Southampton (UK), with distinct surveillance strategies. We showed that the model with NFDS better replicates replacement dynamics than a null model without NFDS, and that NFDS likely only acts on part of the *S. pneumoniae* accessory genome. We found consistent estimates for vaccination effectiveness across the different study locations and region-specific genes under NFDS, highlighting the importance of conducting genomic surveillance in each country of interest. By simulating data from the model, we showed that an optimal surveillance strategy prioritises per-sampling sample size over sampling frequency for small sampling budgets.

**Conclusions:**

Our model can be used to predict vaccine replacement dynamics after PCV introduction, and can be easily reapplied to analyse new data from vaccine introductions or new regions. Our model is available in the R package Stubentiger (Studying Balancing Evolution (NFDS) To Investigate Genome Replacement) on GitHub https://github.com/bacpop/Stubentiger.

**Supplementary Information:**

The online version contains supplementary material available at https://doi.org/10.1186/s13073-026-01672-4.

## Background

*Streptococcus pneumoniae* is a human pathogen that causes pneumonia and meningitis, with the highest incidence of disease in children and elderly adults [1]. Pneumococcal Conjugate Vaccines (PCVs), first introduced in 2000 in the United States, successfully decreased the burden of disease and carriage of targeted serotypes [2, 3]. However, PCVs impact intraspecific competition, greatly reducing the fitness of the proportion of serotypes they affect and leading to the replacement of vaccine targeted serotypes by non-targeted serotypes [4, 5].

Traditionally, *S. pneumoniae* epidemiology has focused on serotypes alone, which are considered to be the main determinant of virulence [6] and are defined by the pneumococcal capsular polysaccharide (CPS). However, the variation of the *cps* locus only represents a small proportion of the genetic diversity. Whole genome sequencing has made it possible to investigate the full breadth of pneumococcal genomic diversity, including causes for invasiveness and antibiotic resistance, and allows the definition of closely-related lineages called global pneumococcal sequence clusters (GPSCs) [7]. While serotypes are defined only by the *cps* region, GPSCs are defined by the variation in the whole genome. Importantly, GPSCs and serotypes provide complementary information since many GPSCs contain multiple serotypes and *vice versa* [7]. Hence, serotype replacement can stem both from genetically similar serotypes from within the same GPSC or genetically different serotypes from other GPSCs [8].

The use of genomics has lead to major breakthroughs in microbial surveillance [9]. In the case of *S. pneumoniae*, our understanding of the pneumococcal population dynamics has been advanced by the Global Pneumococcal Sequencing (GPS) project. By September 2025, the GPS project had sequenced 25,997 pneumococcal genomes across 60 countries [10]. It is crucial to collect and leverage genomic surveillance data in order to understand the mechanisms of vaccine-type replacement, why certain serotypes are more successful in replacing vaccine-targeted serotypes, and ultimately, anticipate the future dynamics of *S. pneumoniae* populations. Additionally, the GPS project has informed optimisation of vaccine formulations and closed gaps in genomic surveillance data in countries with high burden of pneumococcal disease [11]. These countries tend to be low- and middle-income countries, where genomic surveillance faces stronger financial constraints [12], empirical use of antibiotics is higher and infrastructure of clinical microbiology labs is sparse, limiting the sample sizes and sampling frequency. Genomic surveillance therefore has the potential to guide the detection of underlying genetic mechanisms that drive ongoing replacement dynamics but requires careful planning in its rollout. A sampling strategy which was prospectively designed such that key questions about vaccine response could be answered would be a significant improvement over retrospectively attempting to answer these questions.

Negative frequency-dependent selection (NFDS) has been proposed as a major driver of vaccine replacement in *S. pneumoniae* [13–15]. NFDS is a type of selection that depends on the frequency of a given trait within a population: it is especially beneficial to have the trait when it is rare and less beneficial when the trait is common [16]. Examples of genes under NFDS in bacteria are antimicrobial resistance genes and bacteriocins.

Mathematical models using NFDS as an evolutionary process have been applied to describe vaccine replacement in *S. pneumoniae* populations [13–15]. These, and similar models, have helped to identify the strength of NFDS, vaccine impact, and immigration of new strains [17]. The same models can make predictions of future dynamics [14, 18]. However, we identified three key problems with previous modelling approaches. Firstly, previous models had been developed to work with specific datasets and were difficult to reuse by non-experts. This prevents public health bodies from reusing them in their own healthcare contexts, where the models could have the most impact. Secondly, former approaches had not compared the utility of genomic surveillance with serotyping or targeted sequencing data. It remained to be investigated whether the more resource-intensive collection of genomic surveillance data was necessary for effective post-vaccination surveillance or whether serotypes or sets of genes could be used as universal markers for NFDS, potentially allowing to transfer knowledge between countries and datasets. Thirdly, the impact of genomic surveillance sample size and frequency on a model’s ability to estimate epidemiological parameters of interest and predict future replacement dynamics remained unclear.

To solve the first problem of re-usability, we implemented a population dynamics model including NFDS, using the odin modelling framework [19]. By separating model code, genomic data input, and fitting procedures, our new model implementation is easy to adapt and re-use. We tested and compared using input of accessory gene data from state-of-the-art, reproducible bioinformatic tools, which we found superior to previous approaches. We applied our model to genomic surveillance datasets not only from the high-income countries US and UK, but also the lower-middle-income country Nepal. To solve the second problem of the utility of genomic surveillance data, we implemented different model versions that used serotyping or whole genome sequencing data. We built upon previous approaches of NFDS model comparison [13], which, combined with our likelihood-based approach, allowed us to perform rigorous model selection. Our likelihood-based approach also made it possible to test how many intermediate-frequency genes were under NFDS, which helped us to investigate the option of targeted sequencing approaches. To solve the third problem of sample size impact, we fitted the model to simulated data to investigate the effect of different surveillance strategies on parameter estimation and prediction, considering a scenario with limited genomic surveillance budget.

## Methods

### Genomic surveillance datasets

Whole genome sequences from a carriage study in Kathmandu (Nepal) were obtained from the European Nucleotide Archive (ENA); the accession numbers can be found in Additional file 1. The isolates from Nepal were collected in Kathmandu in 2009–2019 with 1881 carriage samples in total. Whole genome sequences and metadata for Massachusetts (US) [20], and Southampton (UK) [21, 22] were previously published. The sequences from the US were collected in Massachusetts in 2001, 2003, and 2007 with 616 sequences in total. The sequences from the UK were collected in Southampton in 2006–2012 with 672 sequences in total. All studies collected samples in children only.

### Genomic data processing

Where necessary, genomes were assembled using shovill 1.1.0 using default parameters [23]. Global pneumococcal sequence clusters (GPSCs) were computed using PopPUNK 2.6.0 [24] with the database for *S. pneumoniae* GPS_v4 via poppunk_assign, applying quality control (–run-qc), allowing maximally one distance of zero (–max-zero-dist 1), and merging a maximum of three clusters based on one single isolate (–max-merge 3). To check whether we are missing any genetic diversity that might be important locally but is not represented by the GPSCs, we ran PopPUNK on the Massachusetts dataset without the GPSC database, which we will refer to as local clustering. Clustering into GPSCs resulted in 52 clusters for Massachusetts, local clustering resulted in 62 clusters. Cluster comparison showed high similarity with an adjusted rand index of 0.975 and adjusted mutual information of 0.959. Using an alluvial diagram, we confirmed visually that the agreement between local cluster assignment and GPSCs is very high, as can be seen in Additional file 2: Fig. S1. In addition to our analysis, GPSCs have been previously shown to represent evolutionary cohesive units [24–27]. We therefore confidently continued to use GPSCs, which are consistent between datasets and hence simplify cross-dataset comparisons.

We constructed a consistent pangenome that included all samples from our three datasets, which ensured that gene clusters were compatible across datasets. We first intended to compute separate pangenomes for each dataset and map the resulting gene clusters to each other using the easy-search function of MMseqs2 [28]. However, mapping two separate pangenomes with MMseqs2 gave a very different result than when computing a joint pangenome using pangenome tools, which we show an example of in Additional file 2: Fig. S2. Hence, we decided to compute our consistent pangenome by giving the data from Southampton, Massachusetts, and Kathmandu jointly to the pangenome tools in the following way: ggCaller 1.3.4 [29] was used for gene calling using the SPARC dataset for annotation [30, 31] and with the option --gene-finding-only. Bakta’s protein bulk annotation workflow (bakta_proteins) [32] with default parameters was used for functional annotation and Panaroo v1.5.2 [33] was run with the options --refind-mode off --clean-mode moderate --family_threshold 0.7 --min_trailing_support 2 --trailing_recursive 0 --min_edge_support_sv 2 --edge_support_threshold 0.0 --aligner none. Insertion sequence (IS) elements, which may generate paralogues within the dataset through inserting in multiple sites, were eliminated by removing all genes with functional annotations containing any of ‘transposon’, ‘transposase’, ‘insertion sequence’, or ‘mobile’ (this removed approximately *2.8 %* of genes). We removed IS elements because it remains challenging to correctly distinguish paralogues from orthologues for these genes [34]. IS elements only account for a small proportion of the overall complement of mobile genetic element genes found in pneumococci [25], and would not include any cargo genes that would be likely to be under NFDS.

For the Massachusetts dataset, we compared the PopPUNK output to sequence clusters created with BAPS [35] and clusters of orthologous genes generated with ggCaller and Panaroo to COGtriangles [36]. The comparison was achieved by fitting model versions on all four data processing combinations (BAPS + COGtriangles, BAPS + ggCaller/Panaroo, PopPUNK + COGtriangles, and PopPUNK + ggCaller/Panaroo) and comparing the resulting likelihoods.

### Processing data for model input

We separated the pangenome matrix (output of Panaroo) for the three countries. Each of these pangenome matrices was then filtered for intermediate-frequency genes, i.e. we only kept gene entries that were present at $5\text {--}95\,\%$ frequency within each population. We made the assumption that the genetic variation (presence or absence of a gene) within the same GPSC is negligible. We therefore summarised the pangenome matrix, which states the presence/absence of genes for each isolate, for the GPSCs. For this, we took the median of all presences (i.e. if over $50\,\%$ of the isolates of a GPSC have a certain gene, the gene would be marked as present, otherwise as absent). This is a 0/1-based approach, since we do not consider intermediate within-GPSC gene frequencies. We also considered using the mean presence of genes within a GPSC, which allows within-GPSC gene frequencies anywhere between zero and one. The mean-based approach resulted in a very similar negative log-likelihood of *-212.09* (compared to *-216.62* for the 0/1-based approach) and similar Bayesian Information Criterion (BIC) of 449.88 (compared to 458.93 for the 0/1-based approach) for the Massachusetts dataset. The posterior distributions of the parameters were also similar, as can be seen in Additional file 2: Fig. S3. We therefore decided to continue using the 0/1-based approach. However, that is not to say that a mean-based approach would not work.

At the beginning of each simulation, the GPSC-serotype combination counts are represented using a matrix, which we refer to as the start population. We generated the start population for the model by Poisson sampling from the GPSC-serotype counts from the first (Southampton and Massachusetts) or first three (Kathmandu) time points. All GPSC-serotype combinations that do not appear in the first time point, but are present at later times, were initialised with one. This is crucial for the correct model population dynamics as the model does not contain a mechanism for creating ‘new’ GPSC-serotype combinations, meaning that combinations that do not exist in the beginning would never occur in the model. We tested the robustness of our approach of initialising these combinations with one in two ways: We compared simulation results of initialising the values with one compared to keeping the values at zero. Additional file 2: Fig. S4A illustrates that the population initialised with zero diverges from those initialised with one, emphasising the need for initialising the values with one. We also compared initialising the values with three and ten, which showed very similar trajectories to initialising with one, demonstrating model robustness to the initial values, as shown in Additional file 2: Fig. S4B.

In the model, GPSCs and serotypes immigrate to the population. The possible GPSC-serotype combinations are defined by the immigration matrix. We calculated the immigration matrix by assigning all GPSC-serotype combinations that appear at least once in the dataset equal probabilities of immigration (i.e. one over the number of GPSC-serotype combinations). The total population size of the model is constant and defined as 15,000 isolates. To test the robustness to the model population size, we fitted the model additionally on estimated region-specific population sizes (details see *Simulating data from the model*). The posterior distributions between model fits on the general model population size of 15,000 and the region-specific population sizes showed remarkable agreement, as shown in Additional file 2: Fig. S5. We hence continued working with the general population size of 15,000.

The delta statistic, as introduced by Corander et al. (2017) [13], calculates the changes in gene frequencies over time. We calculated it in the same way as described in their paper, by calculating the changes in gene frequencies across loci *l* of the first time point (Southampton and Massachusetts) or first three (Kathmandu) *e*, which are assumed to be at equilibrium, to the gene frequencies of the last time point *f*. Genes that change the least in frequency have a low delta statistic value, while genes that change more have a higher value. For gene *l* the delta statistic is defined as 

$$\begin{aligned} \textrm{delta}(l) = & \ \frac{(f_l - e_l) ^2}{1 - e_l (1 - e_l)} \end{aligned}.$$      

For convenience, we then ranked the delta statistic values for all genes. We calculated the ranked statistic independently for Massachusetts, Southampton, and Kathmandu.

Other inputs to the model include a vector that defines which serotypes are affected by the vaccine and when the vaccine rollout started, both of which can be typically found in the literature. The model also needs the information of how many generations of *S. pneumoniae* to simulate between two consecutive data sampling time points. Here, one generation spans the time between two transmission events, which had previously been estimated to be roughly a month for *S. pneumoniae* [17, 37–39]. Hence, we assumed throughout all analyses that there was one *S. pneumoniae* generation per month, meaning that there would be twelve generations per year. This should be given in the form of $dt = 1 / \text {generations between sampling time points}$, e.g. *dt = 1 / 12* for annual sampling.

### Compartmental population dynamics model

Our population dynamics model was based on the model from Corander et al. (2017) [13], which is an extension of the Wright-Fisher model to include immigration, and selection due to vaccination and NFDS. While similar, our model is formulated as a compartmental model rather than an individual-based model. We therefore assume that no novel genotypes can arise during simulation, as this would lead to a variable-dimensional state space, which makes inference numerically challenging.

All model versions of our population dynamics model expect the following data-informed inputs described in the section above: the GPSC-summarised pangenome matrix, start population, immigration matrix, delta ranking, vaccine vector, vaccine rollout time point, and *dt*.

In the following, we will describe the *partial NFDS, strain-serotype* model, which has four parameters that can be fitted to data and considers some but not all genes to be under NFDS (*partial NFDS*). Other model versions used for comparison are described below. The *strain-serotype* model represents the population at time *t* as the matrix $P^t$, with rows representing distinct strains (GPSCs) *i=1,…c ,n* and columns representing distinct serotypes *j=1,…c ,l*. Generations of $P^t$ are non-overlapping, and the subsequent generation $P^{t+1}$ consists of the offspring population $B^t$ and immigrating strains $M^t$ calculated from the current generation $P^t$: $P_{i,j}^{t+1} = B_{i,j}^{t} + M_{i,j}^t$.

The immigrating strains $M^t$ are binomially distributed with success probabilities defined by the immigration matrix $m_{i,j}$, which represents all observed strain-serotype pairs in the data, weighted with equal probabilities, and number of experiments defined by the immigration number $\hat{m}^t$. $\hat{m}^t$ is binomially distributed with number of experiments determined by the carrying capacity *K* and probability of success determined by the immigration parameter *m*, which is fitted to data. The carrying capacity *K* is the constant population size of the model population and can be chosen by the user.

$$\begin{aligned} M_{i,j}^t\sim & \ \textrm{Binomial}(\hat{m}^t, m_{i,j})\\ \hat{m}^t\sim & \ \textrm{Binomial}(K, m) \end{aligned}$$

The offspring population $B^t$ depends on the carrying capacity *K*, the non-immigrating force *1 - m*, and the offspring matrix $p_{i,j}$ of the population $P^t$. The offspring matrix $p_{i,j}$ represents the probabilities of transmission to the next generation, depending on the NFDS term $(1 + \sigma _f)^{\pi _{i,t}}$, the number of individuals in each compartment in the parent generation $P_{i,j}^t$, and the vaccination effect $1 - [v_\textrm{time}(t) v_\textrm{type}(j) v]$.

$$\begin{aligned} B_{i,j}^{t}\sim & \ \textrm{Poisson}\left( K \frac{p_{i,j}^t}{\sum _{i=1}^{n} \sum _{j=1}^{l} p_{i,j}^t} (1-m)\right) \\ p_{i,j}^t= & \ (1 + \sigma _f)^{\pi _{i,t}} P_{i,j}^t \bigl( 1 - [v_\textrm{time}(t) v_\textrm{type}(j) v]\bigr) \end{aligned}$$

    

The NFDS term $(1 + \sigma _f)^{\pi _{i,t}}$ consists of the parameter for the strength of NFDS $\sigma _f$ and the deviation from equilibrium gene frequencies for strain *i*, $\pi _{i,t}$. $\pi _{i,t}$ is calculated by subtracting the current gene frequencies *f* from the equilibrium gene frequencies *e*, for all genes $G_i$ that are found in strain *i* and that are in the set of genes under NFDS *S*. *S* is determined by ordering intermediate-frequency genes according to the delta statistic described above and making a cut-off at the proportion defined by parameter $\mathrm{prop}_f$.

$$\begin{aligned} \pi _{i,t} = \sum \limits _{l \in G_i, l \in S} e_l - f_{l,t} \end{aligned}$$

    

The vaccination effect $1 - [v_\textrm{time}(t) \cdot v_\textrm{type}(j) \cdot v]$ consists of function $v_\textrm{time}(t)$, which returns 1 if the model time is at a post-vaccine time point; the function $v_\textrm{type}(j)$, which returns 1 if serotype *j* is a vaccine type; and the vaccination effectiveness parameter *v*, which can be fitted to data.

In summary, the parameters that can be fitted to data are the vaccination effectiveness parameter *v*, the NFDS strength $\sigma _f$, the proportion of genes under NFDS $\mathrm{prop}_f$, and the immigration rate *m*. The *partial NFDS, strain-serotype* model is implemented in our R package Stubentiger (**Stu**dying **B**alancing **E**volution (**N**FDS) **T**o **I**nvestigate **Ge**nome **R**eplacement), which includes functions for simulating and fitting models, as well as an example dataset. It can be installed from GitHub https://github.com/bacpop/Stubentiger [40].

### Alternative versions of the compartmental population dynamics model

Other model versions are specific variations of the *partial NFDS, strain-serotype* model described above.

- In the *no-NFDS, strain-serotype* model, $\sigma _f$ is fixed to 0 and $\mathrm{prop}_f$ is fixed to 1, as described in Corander et al. [13]. This is a null model that has no notion of NFDS.
- In the *general-NFDS, strain-serotype* model, $\mathrm{prop}_f$ is fixed to 1, hence all intermediate-frequency genes are under NFDS, as described in Corander et al. [13].    
- In the *varying-NFDS, strain-serotype* model, which introduces two gene compartments; one under strong NFDS and one under weak NFDS, the strength of which is denoted by the additional parameter $\sigma _w$, as described in Corander et al. [13].
- The *strain* model only contains frequencies of GPSCs and has no concept of serotypes. In this model, a GPSC is affected by a vaccine if the majority of serotypes within a GPSC are vaccine types.
- The *serotype* model only contains frequencies of serotypes and has no concept of GPSCs. For the data-informed inputs, genetic information is summarised per serotype and the likelihood compares the model outputs to serotpe counts instead of GPSC counts.

Data analysis and plotting was done using R 4.4.1 and RStudio 2024.09.0+375. Our population dynamics model was implemented using the R packages odin.dust (version 0.3.13), odin (version 1.5.11), dust (version 0.15.3), and mcstate (version 0.9.22) [19]. The code can be found on GitHub https://github.com/bacpop/NFDS_Model.

### Fitting the population dynamics model to genomic data

The models were fitted using Markov Chain Monte Carlo (MCMC) using the R package mcstate [19]. For MCMC, we defined a multinomial likelihood. We compared our model to GPSC counts for all sampling time points. Our multinomial likelihood takes these counts and compares them to the model values by summing over all serotypes of a GPSC.

$$\begin{aligned} L(\theta | x) \sim \textrm{Multinomial}( x = \text {GPSC counts, } p = \text {modelled GPSC frequency} ) \end{aligned}$$

Because $\sigma _f$ and *m* tend to be close to zero, we fitted the logarithmic value of these parameters, which makes it easier for MCMC to sample. We defined uniform priors on [0, 1] (which for the two transformed parameters $\sigma _f$ and *m* resulted in 1/*x* priors after transformation).

$$\begin{aligned} p(x)= \left\{ \begin{array}{ll} 1,& \text {if } 0 \le x \le 1\\ 0, & \text {otherwise} \end{array}\right. \end{aligned}$$

We ran four chains of the MCMC in deterministic mode with a deterministic particle filter (which takes the mean of the probability distributions in the model), first for 2000 steps and then for an additional 20,000 steps. For Kathmandu, fitting took longer and we therefore ran the chains for 1000 steps and then 10,000 steps instead. We applied the Chi-Squared distribution function (pchisq) in R to execute likelihood-ratio tests for model comparisons, applying Bonferroni correction to compensate for multiple testing. We also calculated the Bayesian Information criterion (BIC) to compare model likelihoods.

To compare the GPSC- and serotype-frequency changes of our model and the data, we assumed that the data was generated through multinomial draws from the underlying population, to allow us to represent uncertainty of the data due to sample size. We then computed $95\,\%$ confidence intervals by calculating the 0.025 and 0.975 quantiles.

### Identifying consistent gene sets under NFDS

For analysing whether the same genes were found to be under NFDS in different datasets, it was crucial to have the consistent pangenome we described above. However, while the gene clusters were consistent, there were differences in gene frequencies between the countries. To be able to compare the sets of genes under NFDS, we only included genes that were at intermediate frequency in all three countries.

As an alternative approach to the delta statistic described above, we employed a genetic algorithm to find genes under NFDS. We used the genetic algorithm implemented in the R Package GA [41]. The genetic algorithm started with a variety of vectors representing all intermediate-frequency genes, which could be affected by NFDS (one) or not (zero) (’the population’). The algorithm then tried to find the best combination of ones and zeros by changing individual values (’mutation’), combining different zero-one vectors (’recombination’), and choosing the best candidates according to the likelihood described above (’selection’). The selection was informed by feeding the chosen NFDS vector into a version of the *partial NFDS, strain-serotype* model that takes an NFDS vector as input and returns the likelihood. The parameters of the model (vaccination effectiveness, NFDS strength, proportion of genes under NFDS, and immigration rate) were fixed.

To test whether the overlap between genes under NFDS of different countries or methods was larger than to be expected at random, we calculated the expected overlap between two (or multiple) gene sets.

$$\begin{aligned} E(\text {genes in overlap at random}) =\ & \text {proportion of genes in A} \\ & \times \text {proportion of genes in B} \\ & \times \text {total number of genes} \end{aligned}$$

### Simulating data from the model

To generate data for the simulation study of genomic surveillance strategies, we simulated the *partial NFDS, strain-serotype* model 20 years forward in deterministic mode, using data inputs and parameter estimates from Massachusetts. We then sub-sampled this synthetic dataset to $0.1 \%,\ 0.25 \%,\ 0.5 \%,\ 1 \%,\ 2.5 \%,\ 5 \%,\ 10 \%,\ 25 \%$ and for annual, biennial, triennial, and quadrennial sampling. The sub-sampling was designed to reflect a fixed ’sampling budget’. For example, a budget of *1 %* of the model population of 15,000 can be realised as 150 annual, 300 biennial, 450 triennial, or 600 quadrennial samples. We then fitted the model as described above and with three independent replicates. After fitting the model, we sampled parameter estimates from the posterior distribution, simulated the model forward with these parameters and computed a cumulative mean squared error (CMSE) as compared to the original simulated data across all 20 simulation years.

Since constant sample sizes are rare in real data, we additionally tested whether varying sample sizes had an influence on the model inference. We sub-sampled the 20 year-long simulated dataset by combining four-year intervals of the five lowest sampling budgets of *0.1 %* to *2.5 %* (reflecting annual sampling). We tested an increasing sampling budget (four years each of $0.1 \%,\ 0.25 \%,\ 0.5 \%,\ 1 \%, 2.5 \%$), a decreasing sampling budget (four years each of $2.5 \%,\ 1 \%,\ 0.5 \%,\ 0.25 \%,\ 0.1 \%$), and then two random permutations of the four-year intervals (random_1 was in order $0.50 \%,\ 1 \%,\ 0.25 \%,\ 2.5 \%, 0.10 \%$ and random_2 in order $1 \%,\ 0.25 \%,\ 0.10 \%,\ 2.5 \%,\ 0.5 \%$). As shown in Additional file 2: Fig. S6, the fits to the varying sampling budget resulted in intermediate-sized credible intervals compared to the lowest sampling budget of *0.1 %* and the highest sampling budget of *2.5 %* included in the varied sampling. This confirmed our expectation because the varied sampling reflects an intermediate number of total samples, with an average sampling budget of *0.87 %* per year and because larger sample sizes have a stronger influence on the likelihood, conferring model robustness to varying sample sizes. The different ordering (increasing, decreasing or the two random orders) only had a limited effect on the quality of the parameter estimates, with the decreasing sampling budget showing slightly larger credible intervals for the NFDS strength and the proportion of genes under NFDS.

We then estimated which of the constant sampling strategies best reflected the datasets from Massachusetts, Southampton, and Kathmandu. In Massachusetts, samples were collected triennially spanning seven years, equivalent to *616 / 7 = 88* annual samples on average, reflecting a sampling budget of *88 / 15000 = 0.0059* or approximately *0.5 %* of the model population. In Southampton, samples were collected annually spanning seven years, with *672 / 7 = 96* on average, reflecting a sampling budget of *96 / 15000 = 0.0064* or approximately *0.5 %* of the model population. In Kathmandu, samples were collected roughly biennially spanning eleven years, equivalent to *1881 / 11 = 171* annual samples on average, reflecting a sampling budget of *171 / 15000 = 0.0114* or approximately *1 %* of the model population.

These values reflect the sampling in relation to the model population size of 15,000; we also estimated how the sampling compared to the actual bacterial population size in each of our study locations. The actual bacterial population sizes can be approximated by estimating the host population size (here, children under ten years old) and the carriage rates of that population. For Massachusetts, Corander et al. (2017) [13] had estimated a bacterial population size of 200,000, based on an estimated under ten population of 828,129 in 2000 [42] and approximately *25 %* carriage in the population. An average of 88 annual samples then correspond to *88 / 200000 = 0.044 %* sampling of the actual bacterial population. For Southampton, Corander et al. (2017) [13] had estimated a bacterial population size of 50,000, based on an under ten population of 202,404 in 2011 [43] and approximately *25 %* carriage in the population. An average of 96 annual samples then correspond to *96 / 50000 = 0.19 %* sampling of the actual bacterial population. We then estimated the bacterial population size for Kathmandu in a similar fashion: The under ten population in Kathmandu was *111600 + 137162 = 248762* in 2011 [44]. We assumed a slightly higher carriage rate of 1/3, as found in other lower-middle-income countries, such as Tanzania [45]. This resulted in an estimated bacterial population size of approximately 82,921. With 171 annual samples, this related to $171 / 82921 \approx 0.2 \%$ sampling of the actual bacterial population.    

## Results

### A reusable, extensible population dynamics model for analysing genomic surveillance data for vaccine-replacement dynamics in *S. pneumoniae*

**Fig. 1:**
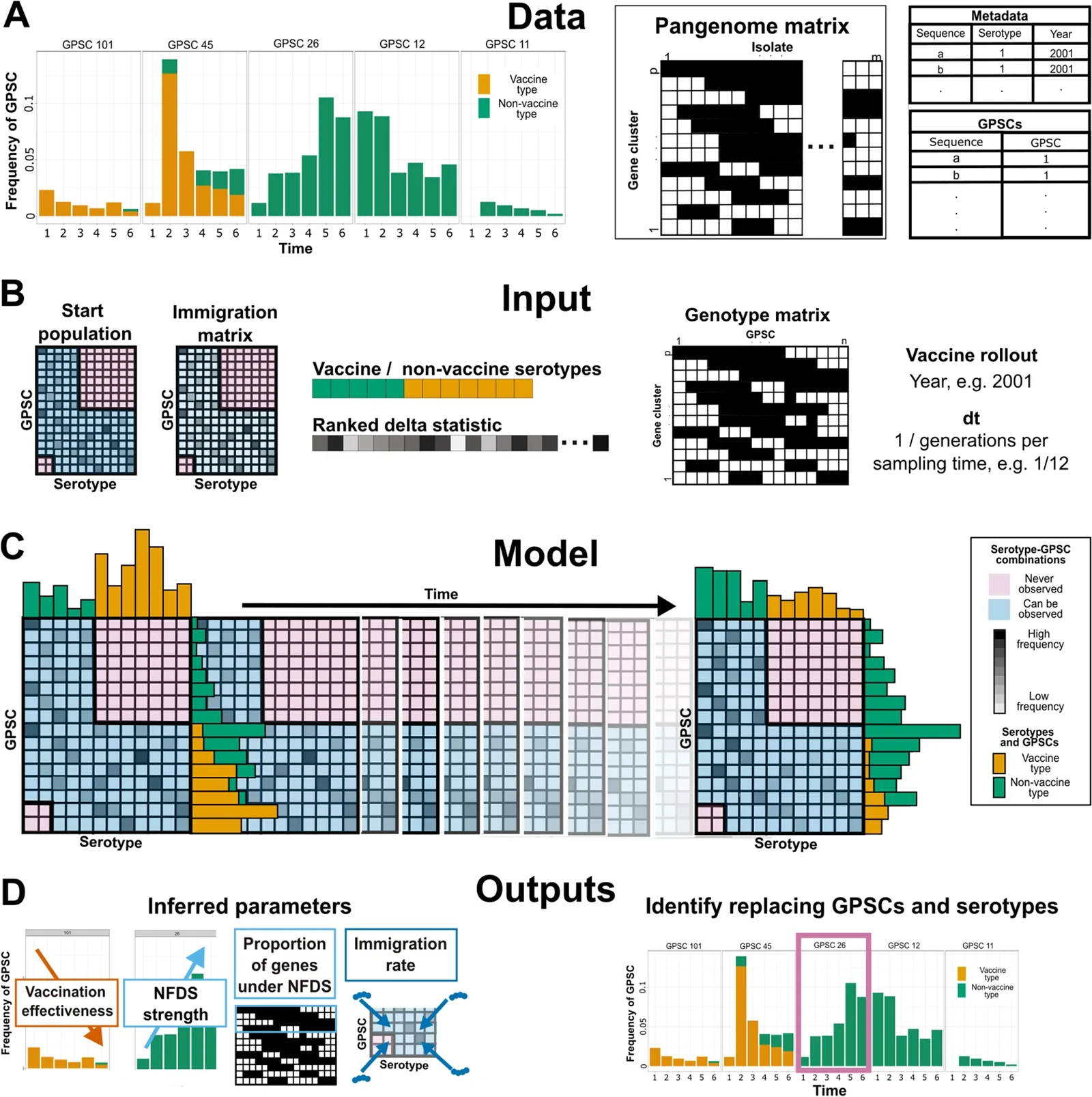
*S. pneumoniae* surveillance data, input, model structure, and model outputs. **A** The Data panel shows illustrative frequencies of *S. pneumoniae* GPSCs, a pangenome matrix, a metadata and GPSC data table. **B** The Input panel shows the data-informed inputs for the model: the start population, the immigration matrix, the vaccine-type vector, the ranked delta statistic, the genotype matrix, the year of the vaccine rollout, and model generations per sampling time. **C** The Model panel shows the two-dimensional Wright-Fisher model structure, representing GPSCs and serotypes. Marginal frequencies represented as bar heights show serotype and GPSC frequencies, respectively, and can be compared across time points. The colours of the bars represent vaccine types and non-vaccine types. **D** The Outputs panel illustrates the four parameters estimated by the *partial NFDS, strain-serotype* model and that replacing GPSCs or serotypes can be identified using the model

Our model describes the population dynamics of *S. pneumoniae* as a function of vaccination, negative frequency-dependent selection (NFDS), and immigration of global pneumococcal sequence clusters (GPSCs) and serotypes. Model outputs are serotype/GPSC density for each modelled time point. Our model is designed to take genomic surveillance carriage data, spanning at least one vaccine introduction, as input, to which the model can be fitted to infer parameters (Fig. 1A). The data, input, model structure, and outputs are shown in Fig. 1.

The population dynamics model is a compartmental Wright-Fisher model with selection. The compartmental structure of the model is two-dimensional, one axis represents GPSCs and the other axis represents serotypes (Fig. 1C). The code outputs the modelled GPSC-serotype frequencies for any time step, as well as the GPSC or serotype frequencies separately (Fig. 1D). In our model the number of compartments is fixed, which makes it less flexible than an equivalent individual-based model but faster to simulate from and fit.

The model is implemented in the domain-specific language odin [19]. Odin has a very similar syntax to R and can be used in RStudio, making it more easily extensible than bespoke compiled code. Furthermore, model training and inference is fast because model code is transpiled to C++, leveraging its runtime advantages. In odin, the model code is separate from the model simulation and fitting, which makes it easy to share models and run them separately from these components. Odin also allows the user to switch between stochastic and deterministic model simulation, which means users can switch between more realistic simulations and faster, deterministic ones.

We designed our model to be used with standard genomic surveillance carriage data of *S. pneumoniae*, which is collected by taking nasal swabs from healthy children across a number of years, followed by culture and whole genome sequencing. To observe dynamics away from endemic equilibrium, samples should ideally be taken before and after vaccine introduction. It is necessary that the data includes the time of sampling. The following data processing steps need to be taken before the data can be given to the model:

1. Sequences should be grouped into GPSCs using PopPUNK [24], which represent genetically similar groups and allow cross-dataset comparisons because GPSCs are consistent.
2. The serotype of each sequence must be determined, which can be done in a laboratory or using bioinformatic tools, like SeroBA(v2.0) [46]. Both determining GPSCs and serotypes can be easily done using the GPS Pipeline [47].
3. The accessory genome must be determined. A good option is ggCaller [29] for calling genes and Panaroo [33] for building a pangenome graph. To input the pangenome matrix into the model, gene presence/absence must be summarised per GPSC since we assumed that within-GPSC variation is negligible. ggCaller and Panaroo are reliable for identifying orthologous genes, which is crucial for correctly estimating multilocus NFDS. We showed that the model fits using ggCaller and GPSCs (from PopPUNK) are equivalent or better than fits based on former state-of-the-art COGtriangles [36] and BAPS [35], which are no longer maintained or need more manual curation, in Additional file 2: Table S1 and Additional file 2: Fig. S7.

The data then needs to be converted to reflect the input format for the model. This is illustrated in Fig. 1B and described in the Methods section.

The model can be fitted to surveillance data with Markov Chain Monte Carlo (MCMC) using mcstate [19]. MCMC estimates a posterior distribution for each parameter value, giving estimates and uncertainty, and allowing for incorporation of priors. Model comparison can be achieved using the model likelihood.

The outputs of our model can help to understand the ongoing vaccine replacement dynamics. They reflect the population dynamics over time and can help to identify replacing GPSCs and serotypes. Similar models have been used to explore the impact of different vaccine designs [18]. The model outputs also include inferred parameter estimates: first, the vaccination effectiveness *v*, which consists of vaccine effectiveness and the coverage of the vaccine rollout, and describes how much the fitness of vaccine types is reduced by the vaccine per generation; second, the NFDS strength $\sigma _f$, which measures the impact of NFDS on the genes which are affected by NFDS, and which confers a frequency-dependent fitness effect per generation on carriers of these genes; third, the proportion of genes under NFDS $\mathrm{prop}_f$, which determines how many of the intermediate-frequency genes are affected by NFDS; and fourth, the immigration parameter *m*, which describes how many members of an external population enter the internal population per generation. Each generation spans one month, meaning that parameters *v*, $\sigma _f$, and *m* should be interpreted as vaccination effectiveness, NFDS strength, and immigration per month. All four parameters also exist in the NFDS model by Corander et al. (2017) but their best-fitting model includes an additional parameter, the “weaker NFDS strength” [13]. This parameter did not improve the fit of our population dynamics model, which we show below. Because the population dynamics model considers NFDS to have an effect on some but not necessarily all intermediate-frequency genes, we will refer to this model as the *partial NFDS, strain-serotype* model.

### Analysing vaccine-replacement dynamics across different countries and vaccination schedules

**Fig. 2:**
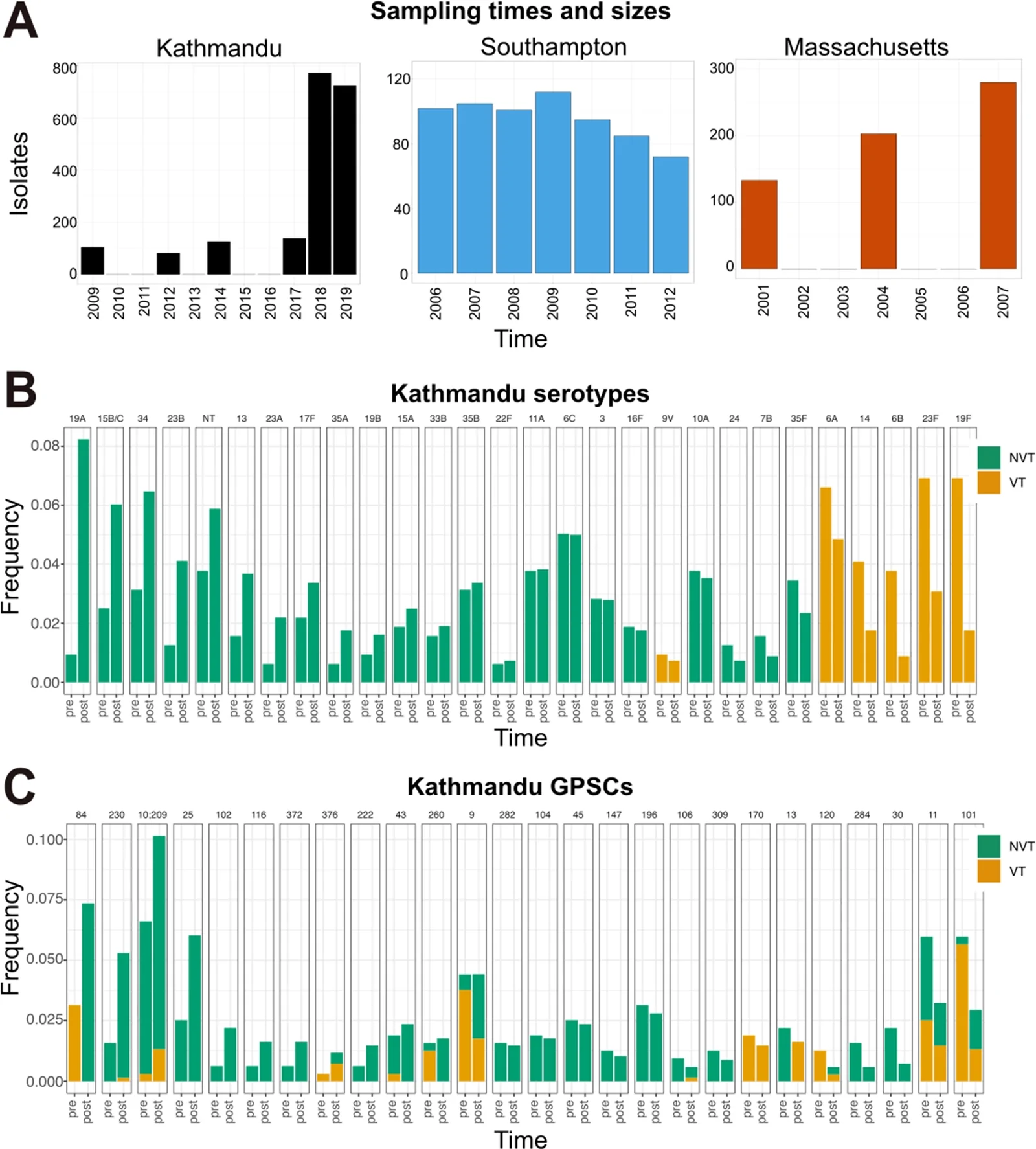
Sample sizes for Kathmandu, Massachusetts and Southampton, and pre- and post-vaccine serotype and GPSC frequencies for Kathmandu. **A** Sample sizes and sampling years for Kathmandu, Southampton, and Massachusetts. **B** Serotype and **C** GPSC frequencies for pre-vaccination time points (mean of 2009, 2012, and 2014) and last post-vaccination time point (2019), sorted from largest positive change to largest negative change, for all serotypes and GPSCs that are present at pre-vaccination samples. Non-vaccine types (NVTs) shown in green and vaccine types (VTs) shown in orange

We studied vaccine replacement dynamics in genomic surveillance data from Massachusetts (US), Southampton (UK), and Kathmandu (Nepal). These three datasets represent different surveillance strategies and introduction schedules of different PCV formulations. The dataset from Massachusetts covers the introduction of the first vaccine PCV7 (affecting serotypes 4, 6B, 9V, 14, 18C, 19F, and 23F), and the sampling was done with increasing sample sizes and consistently every three years (Fig. 2A, right). The dataset from the Southampton covers the introduction of both PCV7 and PCV13 (affecting serotypes 1, 3, 5, 6A, 7F, and 19A in addition to those in PCV7), and sampling was done consistently every year (Fig. 2A, middle). The dataset from Kathmandu covers the introduction of PCV10 (affecting serotypes 1, 5, and 7F in addition to those in PCV7, with cross immunity for 6A [48]), and sampling was done less consistently, with varying sample size and sampling frequency but an overall larger sample size and for a longer time (Fig. 2A, left).

Figure 2B and C show Kathmandu’s pre- and post-vaccine frequencies of serotypes and GPSCs, respectively, ordered by frequency changes. Panel B shows that vaccine types reduce in frequency, while non-vaccine types respond variably: some increase in frequency, some are stable. Panel C shows that the GPSC frequencies tend to be more stable. GPSCs containing vaccine types tend to reduce in frequency but can be partially rescued by non-vaccine types in the same GPSC.

**Fig. 3:**
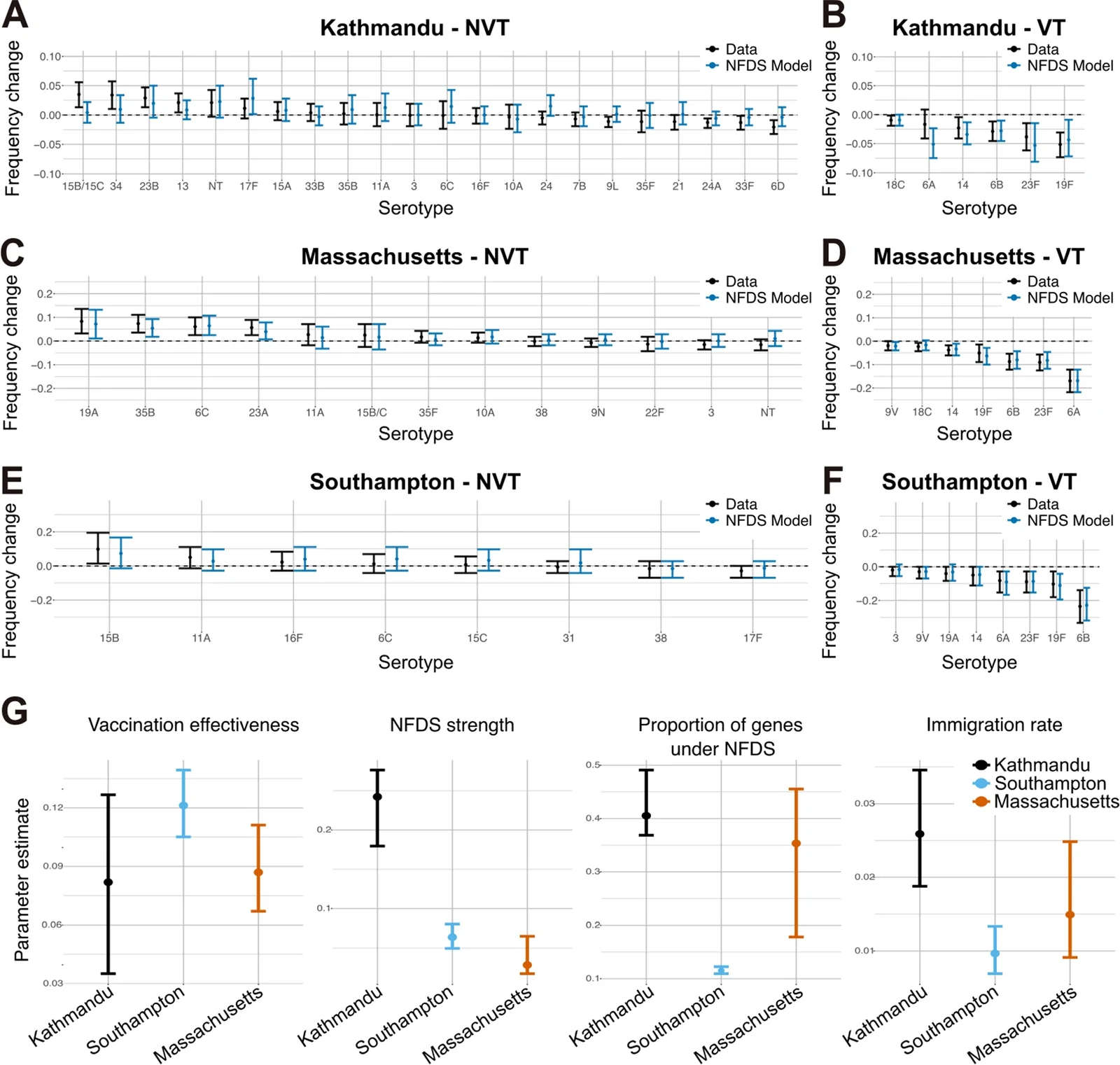
Comparison of *partial NFDS, strain-serotype* model to data and parameter estimate comparison across countries. **A** - **F** Comparison of serotype frequency changes in the data and forward simulation of *partial NFDS, strain-serotype* model with mean and *95 %* confidence intervals. Only serotypes with a frequency of larger than one percent in the pre-vaccine sample are shown. **A** Kathmandu non-vaccine types (NVTs), **B** Kathmandu vaccine types (VTs), **C** Massachusetts NVTs, **D** Massachusetts VTs, **E** Southampton NVTs, **F** Southampton VTs. **G** Parameter estimates for vaccination effectiveness, NFDS strength, proportion of NFDS, and immigration rate with mean and *95 %* credible intervals for Kathmandu, Southampton, and Massachusetts

We fitted the *partial NFDS, strain-serotype* model to each of these genomic surveillance datasets separately. Figure 3A to F compare the changes in serotype frequencies with $95\,\%$ confidence intervals from the data and the *partial NFDS, strain-serotype* model. The plots are split into non-vaccine types (NVTs), A, C, E, and vaccine types (VTs), B, D, F, for Kathmandu, Massachusetts, and Southampton, respectively. When data confidence intervals overlap zero, frequency changes are not significant. Overlapping confidence intervals between model and data can be interpreted as agreement of model and data. All model confidence intervals for all countries overlap with the confidence intervals of the data. The model correctly describes whether the mean frequency of a serotype has increased or decreased for almost all serotypes. Interestingly, for Kathmandu, serotype 6*A*, which is affected by cross-immunity [48], but is not included in PCV10, shows a weaker response to the vaccine in the data. Our model does not capture this effect as we included 6*A* as a standard VT. Similar model fits to Massachusetts and Southampton datasets have been previously reported in Corander et al. (2017) [13], which we used to validate our model formulation and fits. A comparison of model and data GPSC-frequency changes is shown in Additional file 2: Fig. S8.

Figure 3G compares the mean parameter estimates and $95\,\%$ credible intervals of the *partial NFDS, strain-serotype* model across the three datasets from Kathmandu, Massachusetts, and Southampton. The vaccination effectiveness is consistent across all locations and the mean estimate ranges between 0.082 and 0.121. The immigration rate is relatively consistent as well, with its means ranging between 0.0097 and 0.0259. The proportion of genes under NFDS is similar in Massachusetts and Kathmandu but lower in Southampton. The NFDS strength is similar in Massachusetts and Southampton but appears to be stronger in Kathmandu. This is potentially be driven by the within-GPSC replacement in Kathmandu, which was shown in Fig. 2C.

We compared the $95\,\%$ credible intervals for Massachusetts and Southampton of our model fits to the 5-parameter model inference in Corander et al. (2017) [13]. Our $95\,\%$ credible intervals were consistently inside of theirs, with the only exception of the immigration rate *m* in Massachusetts.

### Model comparison shows not all intermediate-frequency genes are under NFDS

Whether all intermediate-frequency genes are affected by NFDS has both ecological and epidemiological implications. It is ecologically relevant because intermediate-frequency genes are expected to sweep or go extinct when there is only drift and directional selection [49]. Investigating whether NFDS alone can explain stable pangenomes is therefore informative. Concerning epidemiology, NFDS might help define gene markers if it only affects a subset of all intermediate-frequency genes, facilitating to monitor GPSC frequencies or to determine gene function and design of future vaccines.

To determine whether NFDS affected all intermediate-frequency genes, we implemented three additional models. The first model was *no-NFDS, strain-serotype* model, which is a null model without the notion of NFDS. It only contains the two parameters of vaccination effectiveness *v* and immigration rate *m*. The second model was the *general-NFDS, strain-serotype* model, which has one additional parameter compared to the *no-NFDS, strain-serotype* model, the NFDS strength $\sigma _f$, which affects all intermediate-frequency genes. Unlike the *partial NFDS, strain-serotype* model, the *general-NFDS, strain-serotype* model does not contain a parameter for the proportion of genes under NFDS; all intermediate-frequency genes are assumed to be affected equally by NFDS. The third model, the *varying-NFDS, strain-serotype* model, has an additional parameter compared to the *partial NFDS, strain-serotype* model, the weaker NFDS strength $\sigma _w$, which affects all intermediate-frequency genes that are not affected by the other NFDS parameter, as determined by the proportion of genes under NFDS parameter. These three models had previously been implemented by Corander et al. (2017) and the authors found the *varying-NFDS, strain-serotype* model to produce the best fits [13].

We fitted all four models to the three datasets and compared the quality of the fits. Both likelihood-ratio tests and Bayesian Information Criterion (BIC) allow a statistical comparison of our models’ likelihoods while considering the number of free parameters (degrees of freedom). The likelihood-ratio test is significant when additional parameters are significantly improving the likelihood and the BIC is lowest for the model version that best explains the data. The *partial NFDS, strain-serotype* model was significantly better than the *no-NFDS, strain-serotype* model and the *general-NFDS, strain-serotype* model, and the *varying-NFDS, strain-serotype* model was not significantly better than the *partial NFDS, strain-serotype* model. The BICs and the significance of the likelihood-ratio test can be found in Table 1.

**Table 1:** BIC and likelihood-ratio test on fits of different model versions. Model versions with degrees of freedom (df) are shown with Bayesian Information Criterion (BIC) and whether they are significantly better (*<0.0001*, ***) than the simpler model versions (with less dfs) according to likelihood-ratio test. The best model version for each country is shown in bold

Model	df	Kathmandu	Massachusetts	Southampton
*no-NFDS, strain-serotype model*	2	2438.25	558.12	1160.00
*general-NFDS, strain-serotype model*	3	$$2297.20^{***}$$	$$544.23^{***}$$	1165.92
*partial NFDS, strain-serotype model*	4	$$\mathbf {2259.65^{***}}$$	$$\mathbf {458.93^{***}}$$	$$\mathbf {952.54^{***}}$$
*varying-NFDS, strain-serotype model*	5	2267.48	465.09	959.07

The result that the *partial NFDS, strain-serotype* model was consistently the best model version demonstrates three things: First, NFDS significantly increases the quality of the fit, suggesting that NFDS plays a role in vaccine replacement dynamics. Second, not all the intermediate-frequency genes identified by Panaroo show signs of being affected by NFDS. While NFDS is thought to strongly contribute to the stability of the accessory genome, it might not be the only factor maintaining the accessory genome. Factors like balanced rates of gene gain and loss or the occupation of different ecological niches might contribute to a stable accessory genome as well [49]. Third, the BIC serves as a test for model overfitting [50]. Hence, the BICs provide evidence against overfitting for the *partial NFDS, strain-serotype* model.

### Whole genome surveillance is required to deploy population dynamics models

Whole genome surveillance is still costly and not available for many past vaccine introductions, which poses the question of whether other data types from surveillance, such as serotyping or targeted sequencing approaches, carry enough information to successfully deploy our population dynamics model.

We first investigated a scenario where only serotyping data was collected but some information about the genetic composition was available (e.g. from other countries or previous studies). To emulate this scenario, we implemented a model version that only contained the frequencies of serotypes (the *serotype model*), summarised the pangenome matrix by serotypes and, in the likelihood, compared the model outputs to serotype counts from the data. The *serotype model* does not fit well to the data, as shown in Additional file 2: Fig. S9. Hence, serotype information alone is not sufficient for deploying our population dynamics model.

We then intended to define a set of genes under NFDS that was consistent across different *S. pneumoniae* populations. Such a gene set could aid vaccine replacement studies because future models would not rely on computing the delta statistics for identifying genes under NFDS, which uses pre- and post-vaccine gene frequencies. This would increase the model’s ability to make predictions. A possible implication would also be to use targeted sequencing as a surveillance strategy, by focusing sequencing efforts primarily on NFDS genes and thereby reducing costs. Potentially, such a gene set could also inform future vaccine development because vaccine replacement dynamics could be understood before the introduction of a new vaccine and proteins that are affected by NFDS could possibly be targeted by serotype-protein vaccines.

**Fig. 4:**
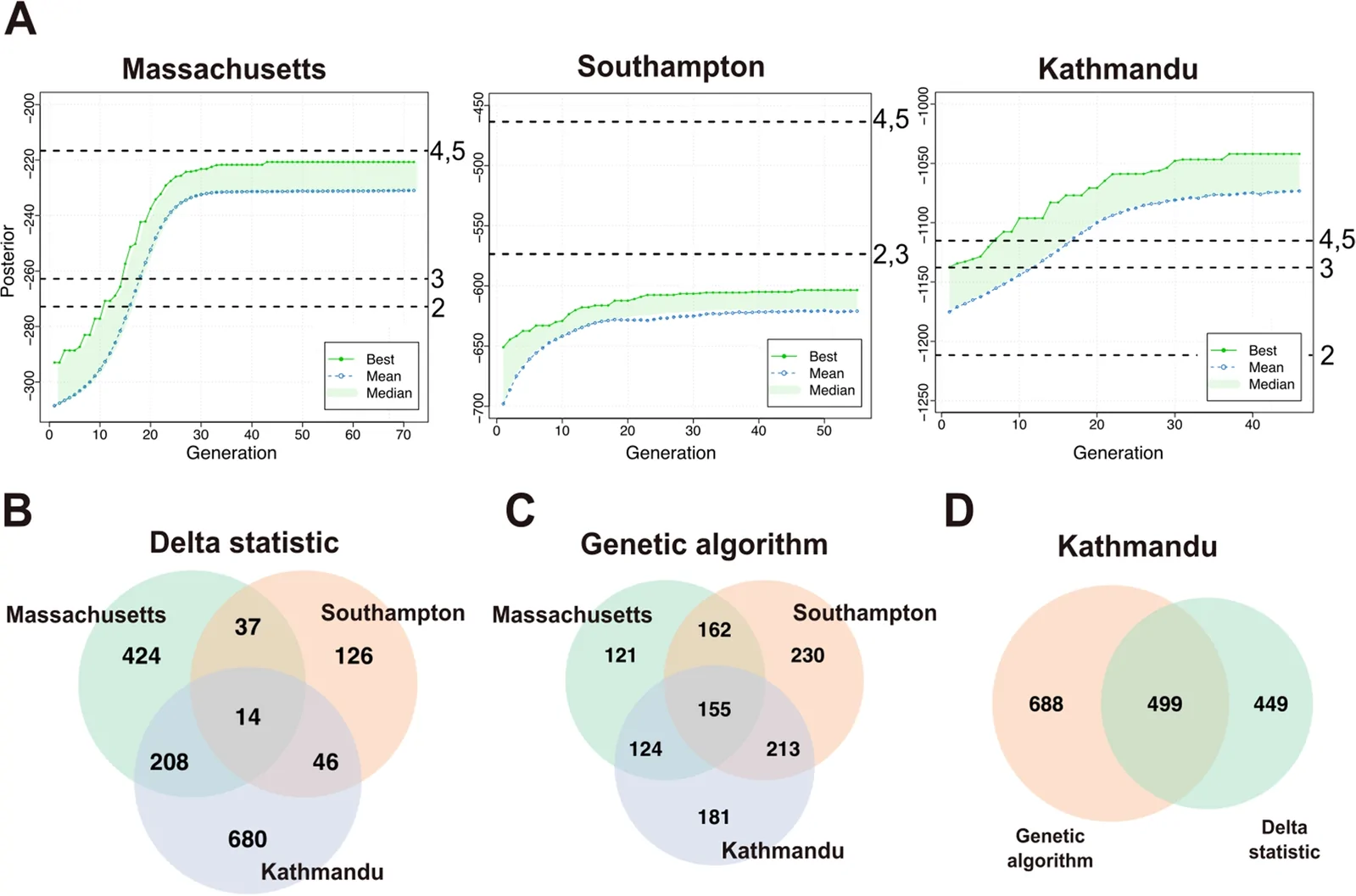
Identifying genes under NFDS using the delta statistic and a genetic algorithm. **A** Likelihood of genetic algorithm for Massachusetts, Southampton, and Kathmandu, and compared to likelihood values of model variants (degrees of freedom 2, 3, 4, and 5) with delta statistic. The x-axis shows iterations of the genetic algorithm (‘generations’). The green shaded area shows the ‘population’ of the genetic algorithm, the green line denotes the best and the blue points denote the average likelihood value. The dashed horizontal lines denote the delta statistic-based fits with 2, 3, 4, or 5 degrees of freedom. **B** Genes under NFDS as determined by delta statistic compared between Kathmandu, Southampton, and Massachusetts. **C** Genes under NFDS as determined with genetic algorithm compared between Kathmandu, Southampton, and Massachusetts. **D** Genes under NFDS in Kathmandu, compared between delta statistic and genetic algorithm

We applied a genetic algorithm to identify such a consistent NFDS gene set. Genetic algorithms are optimisers that are inspired by natural selection. They are typically applied to a ‘population’ of vectors, in which they generate diversity through changing values (‘mutation’) and combining vectors (‘recombination’) and then keep the best vectors according to some fitness function (‘selection’). This process is repeated multiple times (‘generations’). When applying the genetic algorithm, we used the same likelihood as in the other (delta statistic-based) *strain-serotype* models and hence were able to compare these likelihoods directly. Overall, the genetic algorithm performed variably compared to the the delta statistic-based approach. While the genetic algorithm resulted in a better likelihood than the *no-NFDS, strain-serotype* model and *general NFDS, strain-serotype* model but not than the *partial NFDS, strain-serotype* and *varying NFDS, strain-serotype* model for Massachusetts, it resulted in worse likelihoods than any of the delta statistic-based models for Southampton, and did better than all of the delta statistic-based models for Kathmandu, see Fig. 4A.

The conceptual difference between the genetic algorithm and the delta statistic is that the genetic algorithm is purely data-informed. The genetic algorithm aims to find the set of genes under NFDS that maximises the likelihood without any constraints. The delta statistic is based on the assumption that genes that change less in frequency are more likely to be under NFDS. This assumption allows ordering the genes by the delta statistic and means that the delta statistic-based *strain-serotype* models simply determine a cut-off for the proportion of genes under NFDS. For now, both approaches depend on datasets spanning pre- and post-vaccine sampling. However, it is plausible that they capture different signals, potentially making one of them less dataset-specific and allowing us to map information from one dataset to another.

We show in Fig. 4B that 14 genes were found to be under NFDS in all three countries using the delta statistic. The functional annotation of these genes can be found in Additional file 2: Table S2. If the number of genes in the overlap is significantly larger than by sampling three subsets of corresponding sizes randomly from the total set of genes, it is likely that the genes in the overlap have some biological relevance; otherwise they are likely not biologically relevant. The expected overlap at random is 23, which is larger than the 14 genes in the NFDS overlap. However, we hypothesised that the delta statistic might be dataset-specific by design, meaning that small frequency differences might result in a differently ordered gene vector, making it impossible to detect consistent NFDS gene sets.

To test whether the delta statistic hindered us from identifying a consistent NFDS gene set, we applied the genetic algorithm as an alternative method to identify the NFDS gene set. Indeed, the overlap of genes under NFDS between the three countries was 155 genes, many more than in the delta statistic-based analysis; see Fig. 4C. However, since the genetic algorithm estimates a higher proportion of genes under NFDS overall, 155 shared genes was still below the expected number of genes under NFDS at random, which was 158.

The overlap between the genes under NFDS using the delta statistic and the genetic algorithm for Kathmandu is 499, which is slightly larger than the expected 481 genes; see Fig. 4C. This suggests that both approaches are somewhat complementary but might detect similar signals.

Overall, our analysis demonstrates that it is difficult to define a consistent NFDS gene set across different countries and vaccination schedules. This highlights the importance of whole genome surveillance to understand vaccine replacement dynamics, since neither serotyping nor targeted sequencing approaches seem to provide sufficient information for the population dynamics model. The lack of consistency in genes predicted to be under NFDS between different locations also emphasizes the need for genomic surveillance in every country of interest, not just in selected ones.

### Investigating optimal surveillance strategies using model simulations

As NFDS is predicted to affect different genes across locations, we sought to characterise the optimal sample size and sampling frequency to inform the population dynamics model and decrease the uncertainty of parameter estimates, ultimately improving our ability to understand the replacement dynamics. This analysis can be interpreted analogously to power analyses that are commonly used to design clinical studies [51, 52].

We simulated the population dynamics model forward with parameter settings based on the Massachusetts dataset to provide a ground-truth synthetic dataset for comparison. This ground-truth dataset represents the whole *S. pneumoniae* carriage population, rather than a sample. To replicate different sampling strategies, we then sub-sampled the ground-truth dataset by sample size and frequency. In order to compare different sampling strategies, we assumed that there was a fixed and limited ‘sampling budget’, which is represented by the equivalent annual sampling frequency. We assumed that there was a fixed cost per sample and that the sampling frequency did not impact the price of a sample. In other words, a sampling budget of *1 %* represents 150 annual (*1 %* of 15,000), 300 biennial, 450 triennial, or 600 quadrennial samples.

**Fig. 5:**
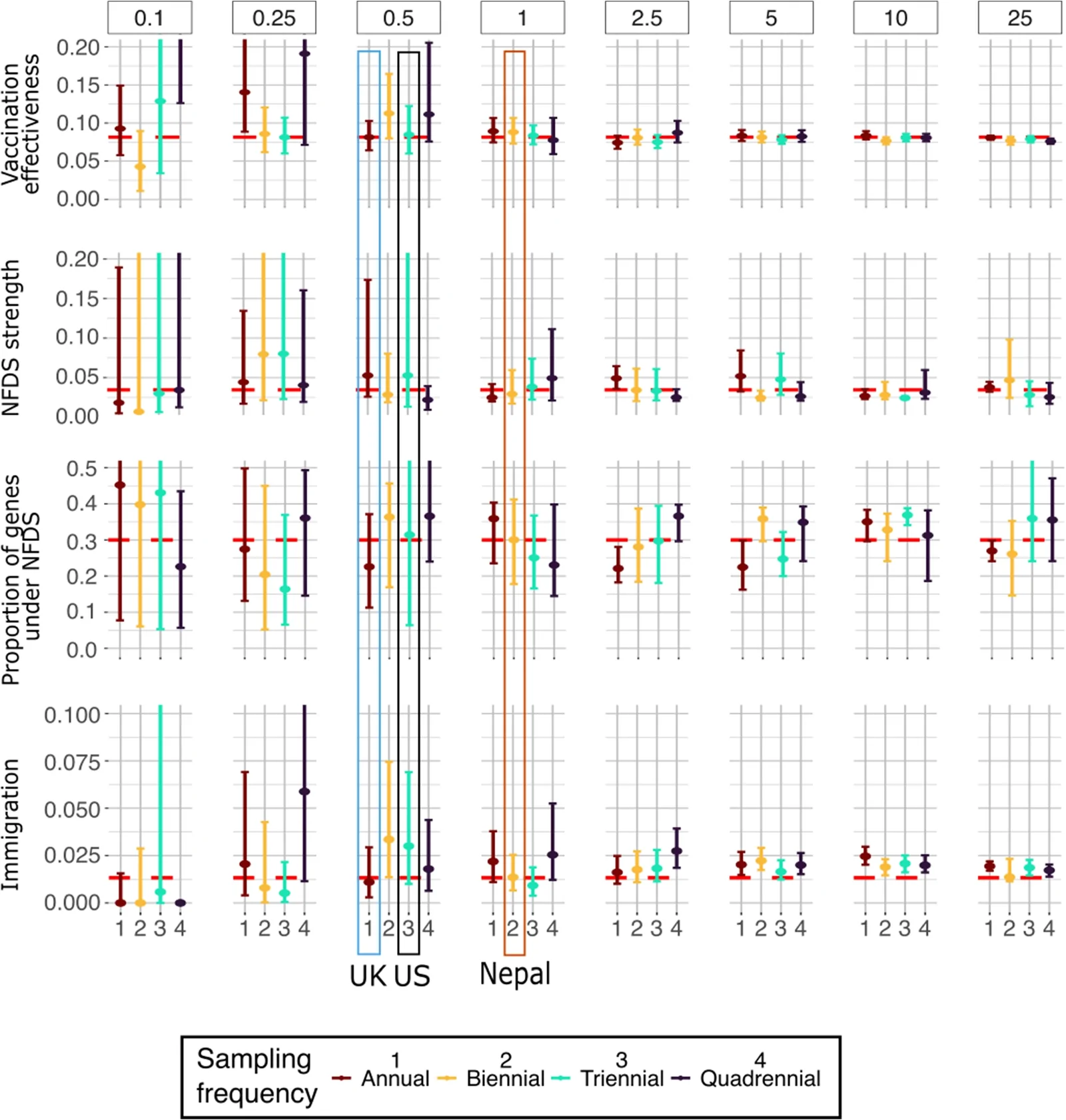
Parameter estimates and credible intervals for fits on synthetic data with different sampling budgets and frequencies. The x-axis and colours show simulated sampling every one, two, three or four years. Y-axis shows parameter values for the four different parameters. Panels show sampling budgets equivalent to sampling *0.1 %* to *25 %* annually of the model population of 15,000 (15 to 3750 annual samples). Original parameter values used to produce synthetic data are shown as red dashed line. Sampling strategies that best reflect sampling in Southampton (UK), Massachusetts (US), and Kathmandu (Nepal), as compared to the model population size, are highlighted. Sampling, as compared to the actual population sizes, was lower: *0.044 %* in Massachusetts, *0.19 %* in Southampton, and *0.21 %* in Kathmandu

We then fitted the population dynamics model to these sub-sampled synthetic datasets and compared the parameter estimates to the true parameter values that were used to generate the simulation; the results of this are shown in Fig. 5. First, the vaccination effectiveness and the immigration rate are easier to estimate than the NFDS strength and proportion of genes under NFDS, which can be seen by the fact that the credible intervals tend to be much smaller for the first these parameters. However, the immigration rate estimation appears to be slightly biased towards larger values. Second, for a larger sampling budget (corresponding to sampling *2.5 %* to *25 %* annually), sampling frequency has little impact on means and credible interval sizes. Third, and surprisingly, for smaller sampling budgets (especially *0.1 %* and *0.25 %* annually) both annual and quadrennial sampling result in biased estimates, whereas biennial and triennial sampling result in better parameter estimates. This might be because the sample size for annual sampling is then very low, lacking the statistical power to detect changes, and the quadrennial sampling might miss critical change points in the data, due to the low sampling frequency.

In Fig. 5, we also highlighted the sampling strategies that most closely reflect the sampling in Massachusetts, Southampton, and Kathmandu datasets. Linking back to the parameter estimates for these datasets in Fig. 3C, the simulation results suggest that the estimates for the vaccination and immigration parameter can be trusted because the credible intervals of the parameter estimates overlap with the true parameter value and the credible intervals are relatively small. The credible intervals on the NFDS strength and proportion of genes under NFDS however show that the exact parameter estimates should be interpreted with caution because they tend to be larger and mean estimates can be biased. To get a reliable estimate for the NFDS strength and proportion of genes under NFDS, much larger sample sizes are necessary.

Figure 5 is useful for evaluating the impact of the sampling frequency on parameter estimation when assuming a constant sampling budget (per panel). Conversely, for evaluating the impact of the sampling budget when assuming a fixed sampling frequency, grouping the data by sampling frequency can be more informative. Hence, we included Additional file 2: Fig. S10, which shows the same parameter estimates but grouped by sampling frequency and demonstrates that higher sampling budget tends to result in better parameter estimates and narrower credible intervals.

**Fig. 6:**
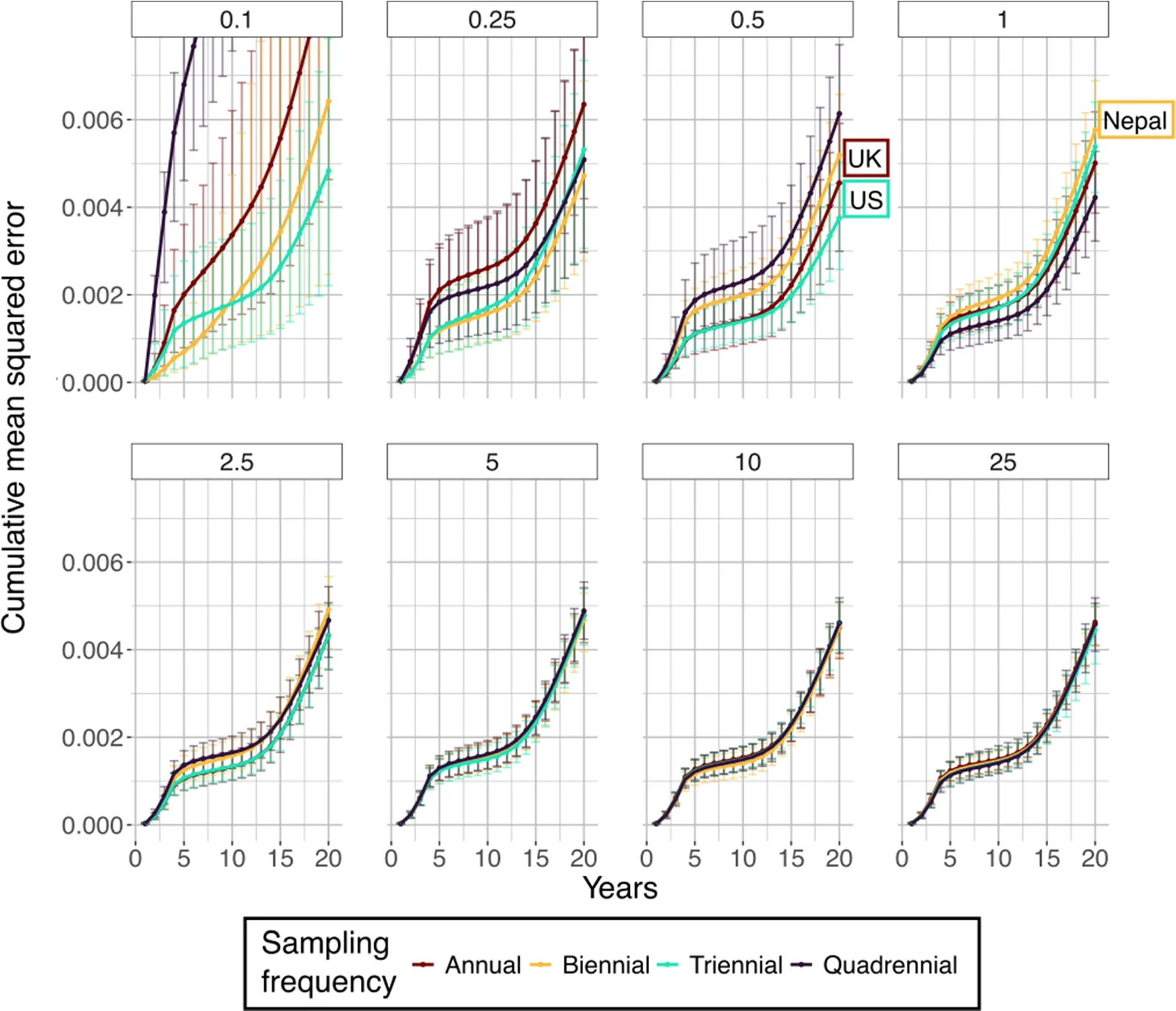
Cumulative mean squared error (CMSE) for model simulation based on fitting parameters on synthetic data with different sample sizes and frequencies. The *x*-axis shows the number of years in the forward simulations. The *y*-axis shows the CMSE. The colours show simulated sampling every one, two, three or four years. The panels show the budget sizes equivalent to annually sampling $0.1\,\%$ to $25\,\%$ of the model population of 15,000 (15 to 3750 annual samples). Sampling strategies that best reflect sampling in Southampton (UK), Massachusetts (US), and Kathmandu (Nepal), as compared to the model population size, are highlighted. Sampling, as compared to the actual population sizes, was lower: *0.044 %* in Massachusetts, *0.19 %* in Southampton, and *0.21 %* in Kathmandu

To answer the question of which sampling strategy results in the best representation of the underlying population dynamics, we used the obtained parameter estimates to simulate the model forward. We then compared those simulations to the ground truth from our synthetic dataset using a cumulative mean squared error (CMSE) across the 20 simulated years. Lower CMSE values suggest better agreement between simulation and ground truth. As depicted in Fig. 6, the lowest CMSEs were achieved with sampling budgets equivalent to *2.5 %* annual sampling and higher. Similarly to our results on the parameter estimation, for low sampling budgets of *0.1 %* and *0.25 %*, better results are achieved by sampling biennially or triennially, rather than annually or quadrennially. Overall, our findings suggest that when trying to make the most of a limited sampling budget, it would be best to focus sampling efforts on biennial or triennial sampling.

## Discussion

The introduction of PCVs introduced new selection pressures to *S. pneumoniae* populations. This can result in unwanted dynamics, such as the growth of the multi-serotype, multi-drug resistant GPSC10 lineage after the introduction of PCV13 [53]. Mathematical models of population dynamics can help reveal and predict post-vaccine replacement dynamics from genomic surveillance data. These models can also help to inform prospective surveillance strategies. Previous work showed successful application of these models to genomic surveillance data but with limited reusability and without investigating how specific sampling strategies might impact the model’s reliability.

We implemented an optimised population dynamics model incorporating NFDS, which we applied to study post-vaccine replacement dynamics in *S. pneumoniae*. By leveraging the modularisation provided by odin, our model is easier to reuse and adapt, uses reproducible bioinformatics tools, and therefore enables its application to new datasets and regions by public health researchers. We applied our model to genomic surveillance data from Massachusetts, Southampton, and Kathmandu. Key findings were that the accessory genes inferred to be under NFDS were not significantly similar across countries, and dynamics could not be predicted by serotype alone. This showed how important region-specific whole genome surveillance is and, hence, we applied our model to investigate optimal surveillance strategies. We showed that for a limited sampling budget it is better to sample less frequently — every two or three years rather than every year — to increase the size of each sample. For larger sampling budgets, sampling every year and every two, three or four years gave equivalent results, so the sampling strategy should be entirely informed by practical considerations.

We showed that modern tools can be used to construct a pangenome in an automated manner, suitable for use in NFDS models. However, it is important to note that gene identification and clustering is difficult, especially across multiple datasets, potentially leading to false positive gene identification, which in turn can lead to issues when estimating the impact of NFDS on loci. Consistent pangenomes across different countries are crucial for comparing gene frequencies and genes under NFDS. Yet, creating consistent pangenomes based on existing, region-specific pangenomes is not straightforward, as we showed in the Methods section. A post-hoc mapping of two separate pangenomes with MMseqs2 [28] gave a very different result than a consistent pangenome computed jointly using ggCaller and Panaroo. In the case of our analysis, we opted for computing a consistent pangenome by doing an analysis on all countries jointly but that might not always be feasible, especially due to runtime and memory requirement on large pangenomes. Hence, developing approaches to join existing pangenomes into a consistent one would be desirable.

Our rigorous model selection suggests that NFDS has an effect on many, but probably not all, intermediate-frequency genes. This finding has implications for studying pangenome dynamics, since stable frequencies of some genes might be retained through other means than NFDS, such as balanced rates of gene gain/loss or niches [15, 49, 54] and because some gene frequencies might be less stable than expected under the influence of NFDS. Disentangling how genes are maintained at stable frequencies also links back to the question of which population genetics mechanisms enable NFDS, which has recently been explored by Guillemet and Lehtinen (2026) [55].

There are many possible ways to extend this model in the future. For example, a factor that we have not explicitly modelled in this study, but strongly influences bacterial population dynamics, is antimicrobial resistance (AMR). Evolutionary mechanisms affecting AMR after the introduction of PCVs and efforts to model the dynamics of AMR in *S. pneumoniae* have been summarised by Watkins et al. (2022) [56]. For example, modelling suggested that longer carriage duration increased the fitness advantage of resistance [57]. Developing a model that explicitly models both AMR and NFDS could be powerful to study post-vaccine dynamics, not least because AMR itself might be under a form of NFDS [13].

Microarray techniques, which can be used for serotyping [58], are being extended to detect GPSCs from plate-sweep DNA [59]. It would also be possible to add probes for specific genes under NFDS. This could be a cost-effective alternative to whole genome sequencing for *S. pneumoniae* surveillance, increasing the frequency and sample size to a degree that enables accurate forecasting. However, this would limit the ability to discover new selection effects, and it may be challenging to create universal arrays for all populations. Future work should therefore include investigating the suitability of new microarray data for estimating replacement dynamics using our model.

We investigated how the model accumulates errors in forward simulations using our synthetic dataset but it remains to be investigated how much predictive power the NFDS model has for *S. pneumoniae* post-vaccine dynamics in real datasets. The limited sampling years we had available and the dataset-specificity of the genes under NFDS did not allow us to investigate this question. However, our model code, which is published in form of an R package, now equips public health surveillance agencies with contemporary data to be able to answer this question in a principled manner.

## Conclusions

We developed a modular, reusable population genetic model that includes parameters for vaccination effectiveness, NFDS, and immigration rates. We showed that our model fits well to genomic carriage data of *S. pneumoniae* from Massachusetts, Southampton, and Kathmandu. Overall, our work demonstrates how crucial it is to do genomic surveillance of *S. pneumoniae* in every country of interest before and after vaccine introduction. We also discovered that in low-budget settings, it can be beneficial to sample less often in order to have high sample sizes per time point and therefore higher statistical power. Our model is available in the R package Stubentiger (**Stu**dying **B**alancing **E**volution (**N**FDS) **T**o **I**nvestigate **Ge**nome **R**eplacement) on GitHub https://github.com/bacpop/Stubentiger [40].

## Supplementary Information

Additional file 1. Kathmandu ENA accession numbers, List of ENA accession numbers for Kathmandu dataset, comma-separated values.

Additional file 2. Supplementary Material, Additional figures and tables, portable document format.

## Data Availability

The whole genome sequences from Kathmandu, Nepal, were obtained from the European Nucleotide Archive (ENA) under project PRJEB3084, the accession numbers can be found in Additional file 1. Whole genome sequences and metadata for Massachusetts, US, were published under project ERP000809 by Croucher et al. [20], and for Southampton, UK, under project PRJEB2417 and PRJEB6332 by Gladstone et al. [21, 22]. The GFFs and output of the joint pangenome analysis with Panaroo were published on zenodo https://doi.org/10.5281/zenodo.20208643 by Lorenz [60]. The analysis code was published on GitHub https://github.com/bacpop/NFDS_Model by Lorenz [61]. The R package Stubentiger is accessible on GitHub https://github.com/bacpop/Stubentiger by Lorenz and Russell [40].

## Abbreviations

AMR: Antimicrobial resistance
BIC: Bayesian Information Criterion
CMSE: cumulative mean squared error
CPS: capsular polysaccharide
ENA: European Nucleotide Archive
GPS: Global Pneumococcal Sequencing
GPSC: Global Pneumococcal Sequence Cluster
NFDS: Negative frequency-dependent selection
NVT: non-vaccine-type
PCV: pneumococcal conjugate vaccine
VT: vaccine-type
