# Supplementary material for: A reusable model of pangenome selection informs optimal surveillance strategies over vaccine introductions
Source: Genome Med. 2026 Sep 7;18:131. doi: 10.1186/s13073-026-01672-4 (PMC13551875; doi:10.1186/s13073-026-01672-4)
Supplement: Supplementary file 1 [file 13073_2026_1672_MOESM2_ESM.pdf]

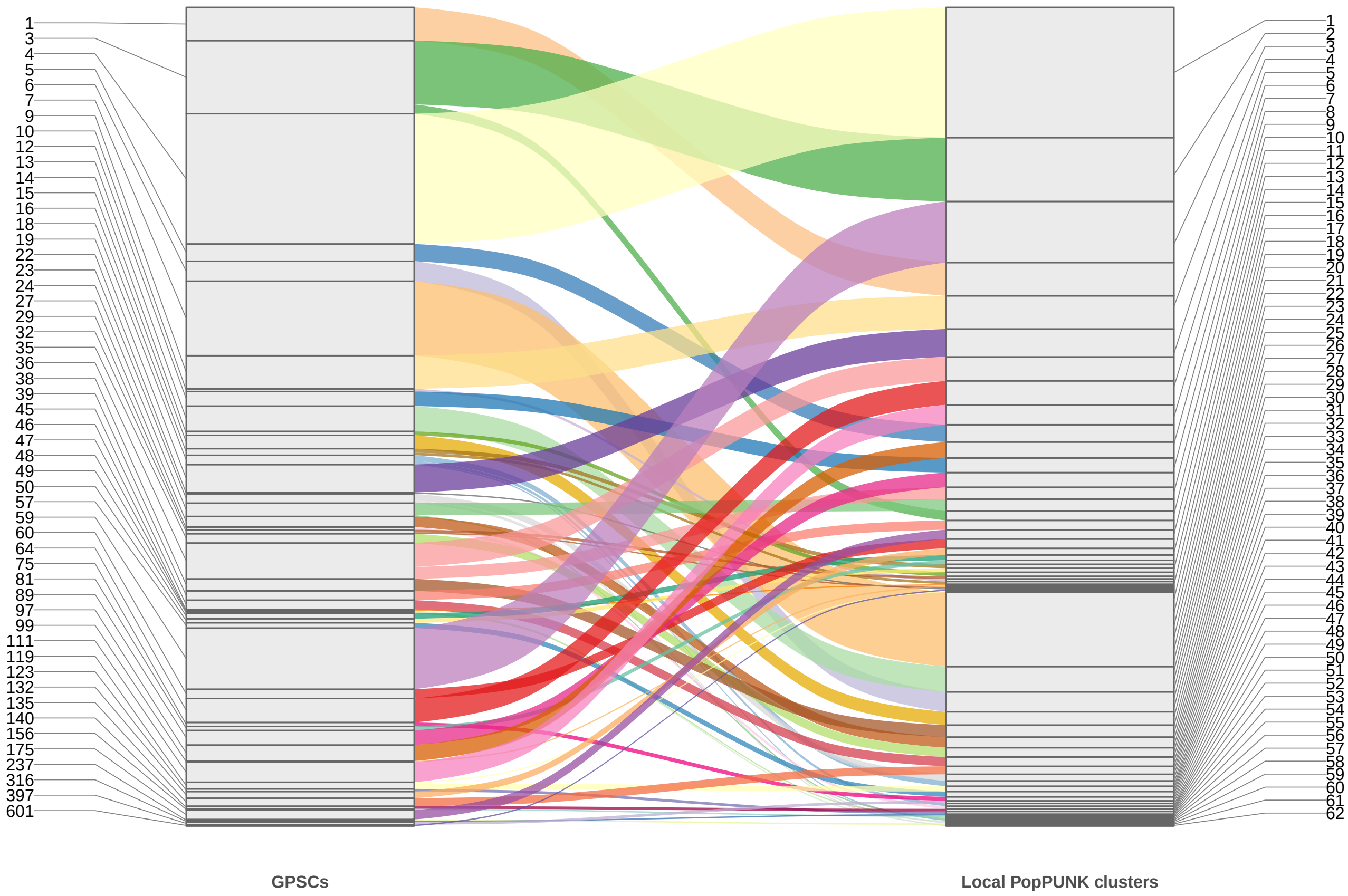

Fig. S1: Comparison of PopPUNK clustering into GPSCs or 'local clusters'.

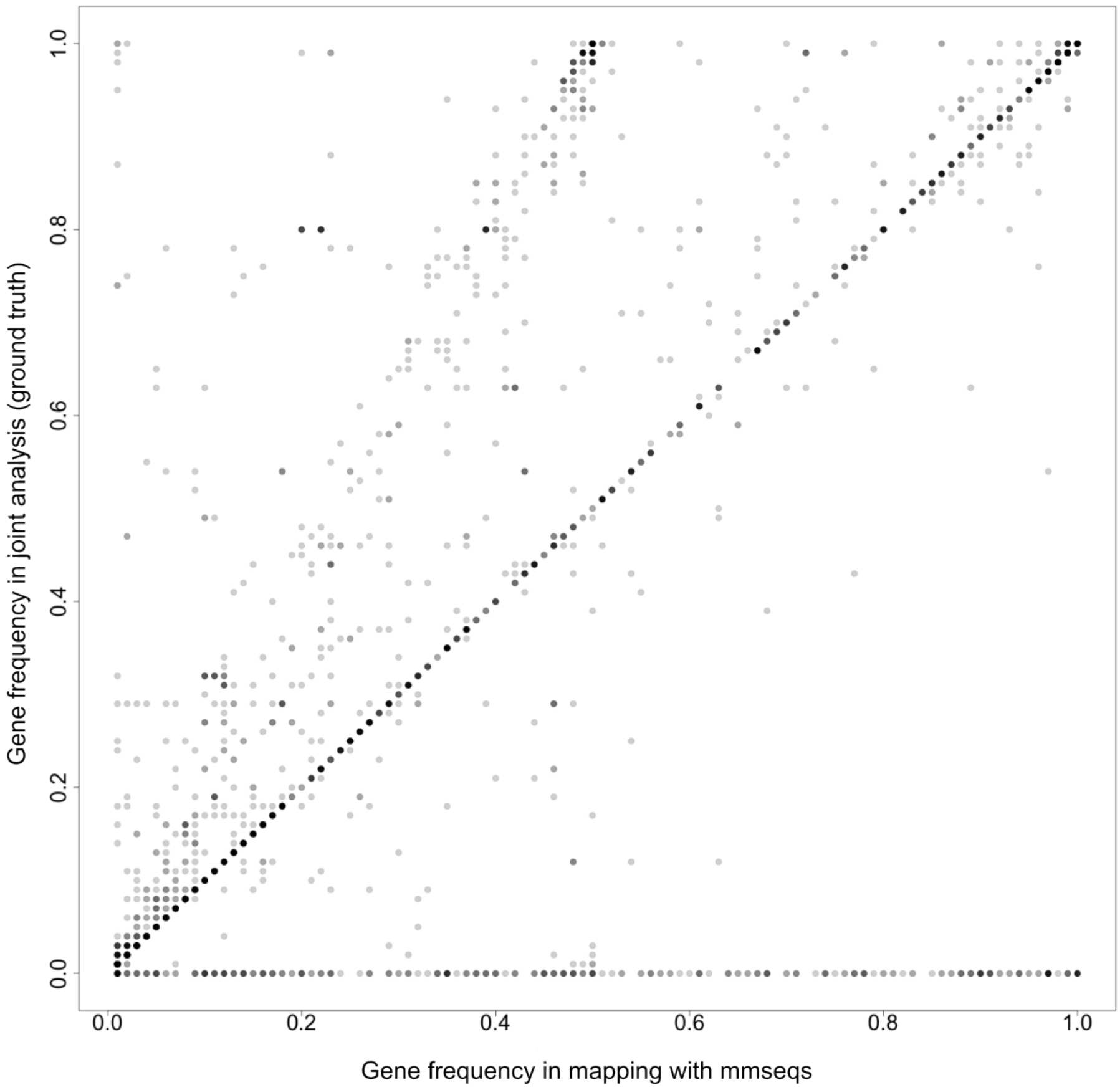

Fig. S2: Comparison of post-hoc pangenome mapping using MMseqs and joint pangenome inference using ggCaller and Panaroo.

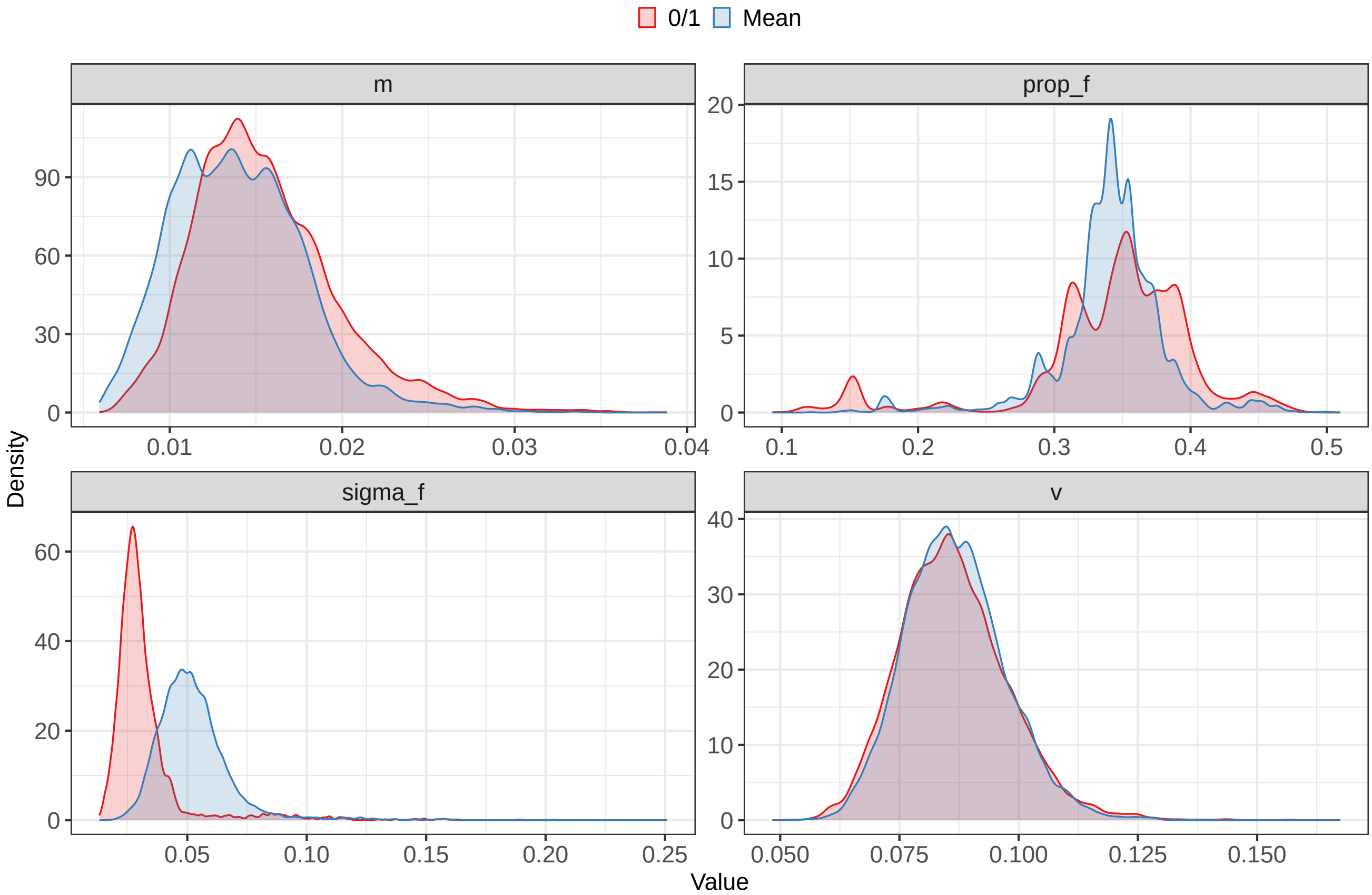

Fig. S3: Comparison of parameter estimates between mean- and 0/1-based model.

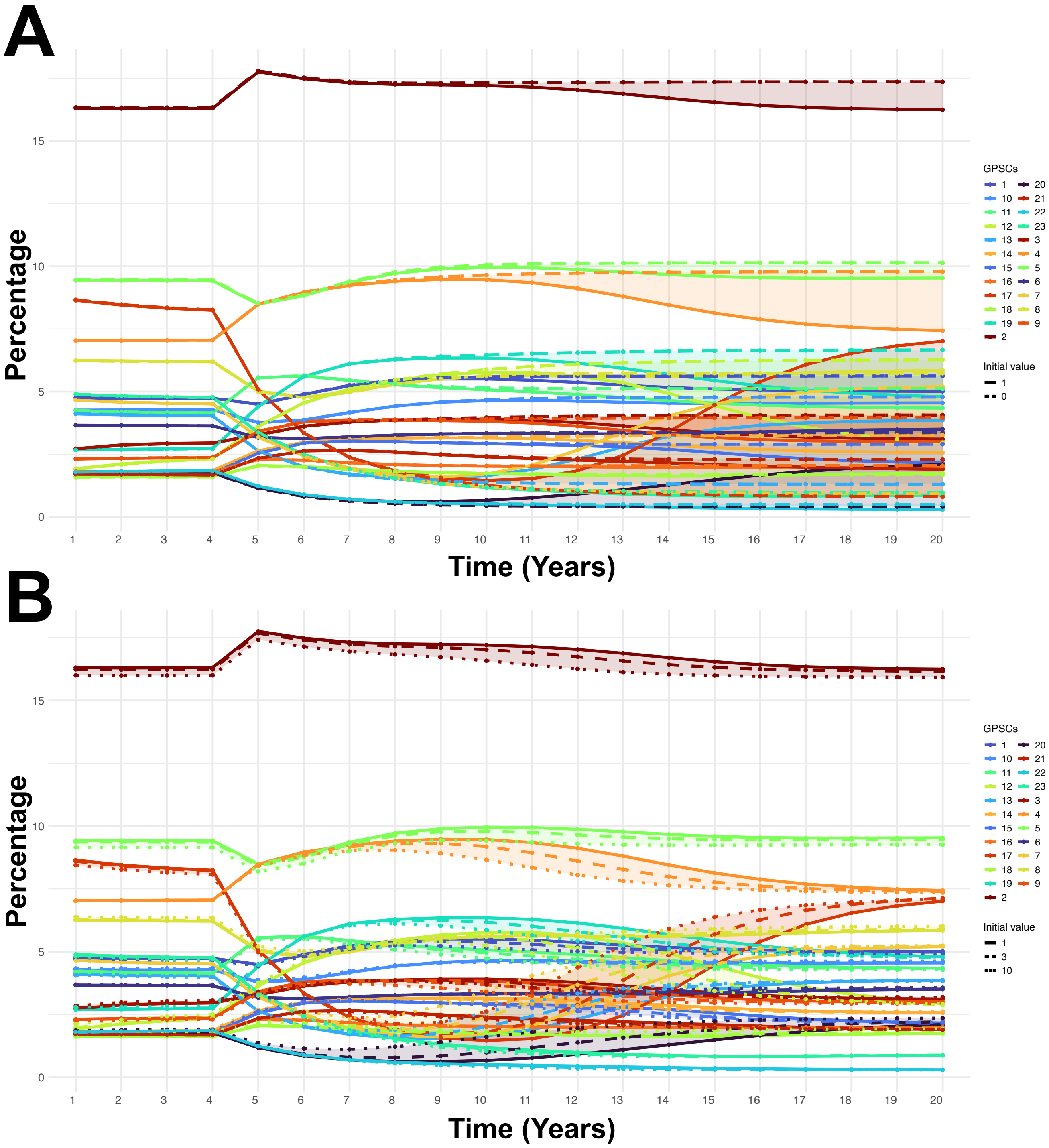

Fig. S4: Robustness of initialising GPSC-serotype combinations, which do not exist in the first time point but later, with one. Forward simulations for 20 years are shown, with the same parameters and inputs as for the other forwards simulations (Massachusetts-based). Line colours represent GPSCs, line types represent initial values. A) Comparison of initialising GPSC-serotype combinations with one or zero. B) Comparison of initialising GPSC-serotype combinations with one, three or ten.

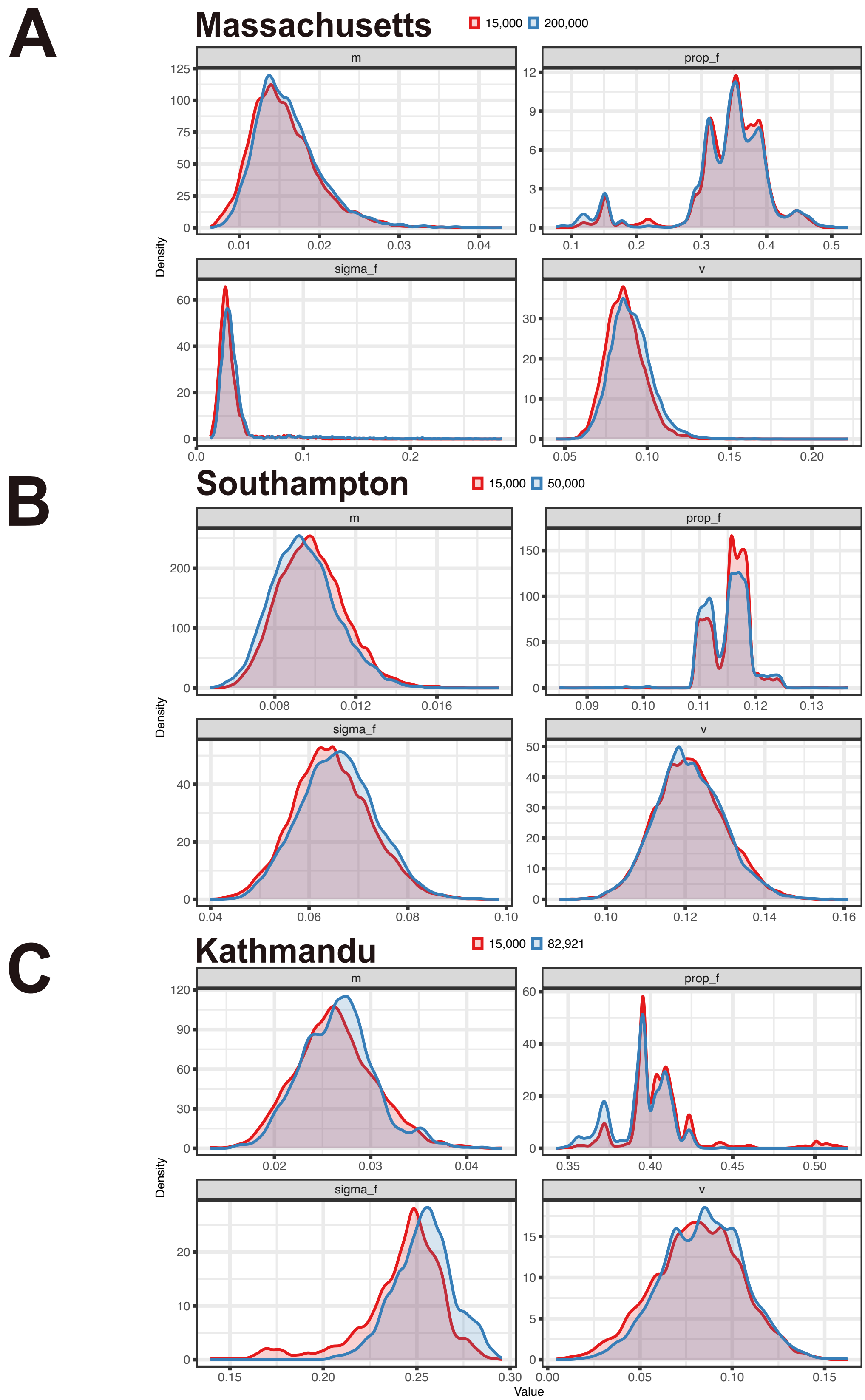

Fig. S5: Comparison of parameter posterior distributions with model population of 15,000 for all populations and region-specific model population sizes based on bacterial population size estimates (see Methods). The region-specific population sizes were A) 200,000 for Massachusetts, B) 50,000 for Southampton, and C) 82,921 for Kathmandu.

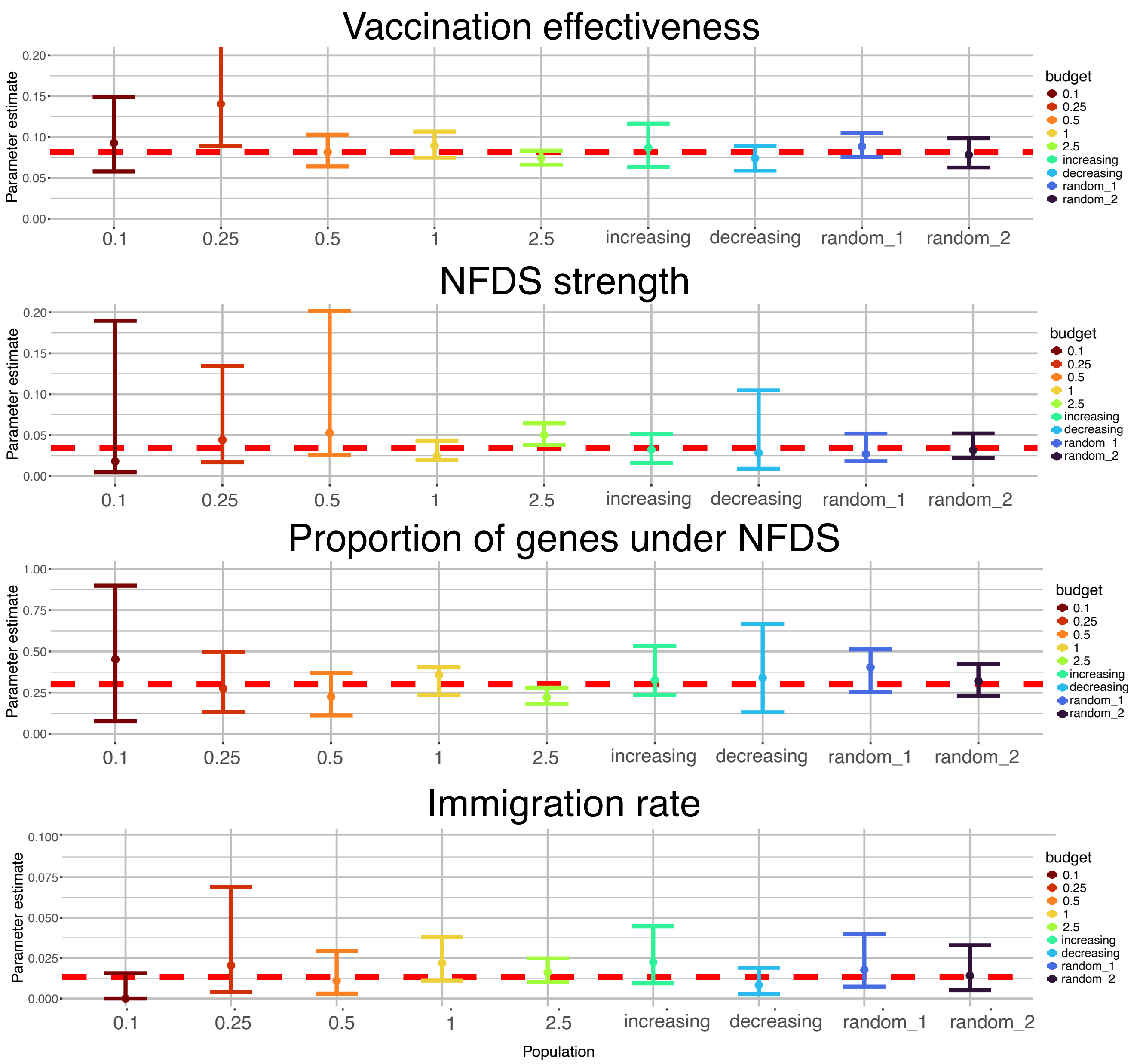

Fig. S6: Parameter estimates and credible intervals for fits on synthetic data with constant or varying sampling budgets and annual sampling. The x-axis and colours show simulated sampling budgets equivalent to sampling 0.1% to 2.5% annually of the model population of 15, 000. Y-axis shows parameter values for the four different parameters. Original parameter values used to produce synthetic data are shown as red dashed line. Sampling strategies closest to the ones in Southampton, Massachusetts, and Kathmandu are highlighted. The model was fitted three times to simulated data, which was downsampled for annual, constant sampling for 20 years for budgets of $0.1\%, 0.25\%, 0.5\%, 1\%, 2.5\%$ of the population. The same was repeated for varying annual sampling budgets of increasing budget (four-year intervals of 0.1% to 2.5%), decreasing budget (four-year intervals of 2.5% to 0.1%), and two random permutations of the four-year intervals (random_1 was in order $0.50\%, 1\%, 0.25\%, 2.5\%, 0.10\%$ and random_2 in order $1\%, 0.25\%, 0.10\%, 2.5\%, 0.5\%$).

Table S1: **Comparison of negative log-likelihoods when using COGtriangles or ggCaller/panaroo and Baps or PopPUNK output.** To make likelihood comparable between approaches, it is computed based on the Baps clusters. Best overall likelihood is highlighted in bold. All values based on fits to the Massachusetts dataset.

Model	COGtriangles + Baps	ggCaller/panaroo + Baps	COGtriangles + PopPUNK	ggCaller/panaroo + PopPUNK
varying-NFDS model	−192.88	−193.10	−188.60	**−179.34**
general-NFDS model	−253.87	−251.72	−250.98	−250.10
no-NFDS model	−251.83	−247.3254	−260.3223	−267.9277

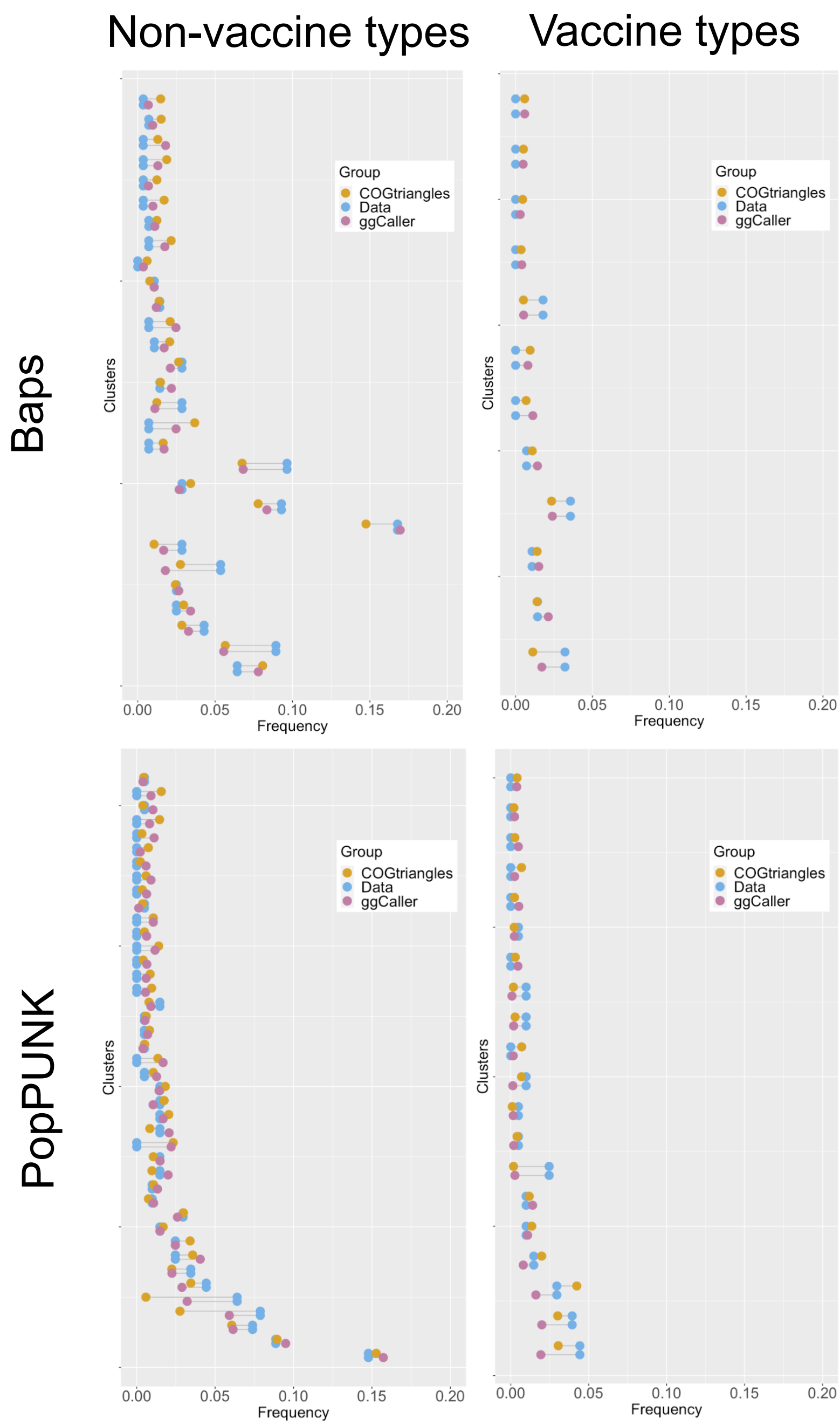

Fig. S7: Visual comparison of fits when using COGtriangles or ggCaller/panaroo and Baps or PopPUNK.

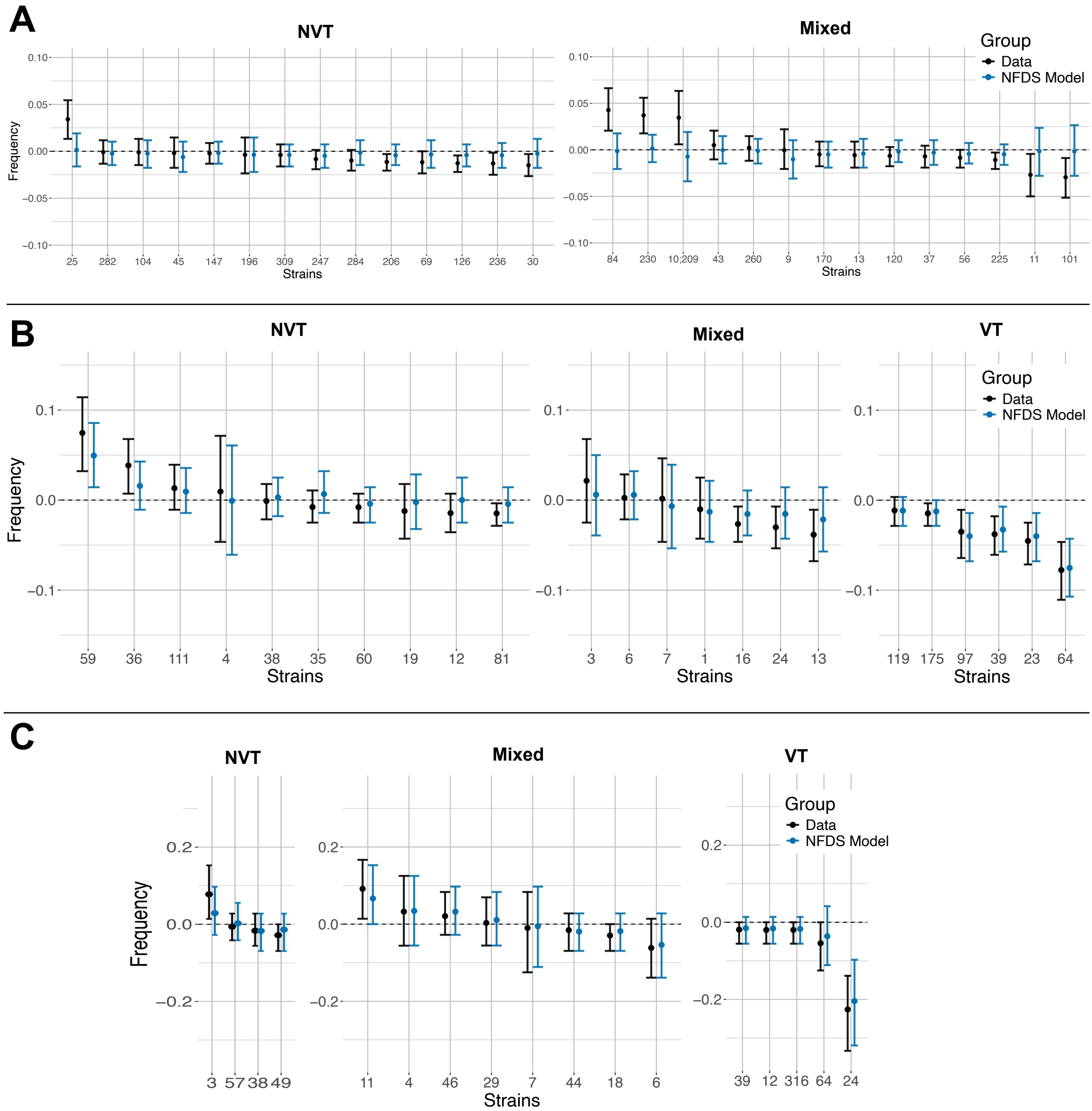

Fig. S8: Comparison of GPSC frequency changes in the data and forward simulation of *partial NFDS, strain-serotype* model with mean and 95% confidence intervals. Only serotypes with a frequency of larger than one percent in the pre-vaccine sample are shown. Plots are split into NVT, mixed, and VT GPSCs. Panels show comparison for A) Kathmandu, B) Massachusetts, and C) Southampton.

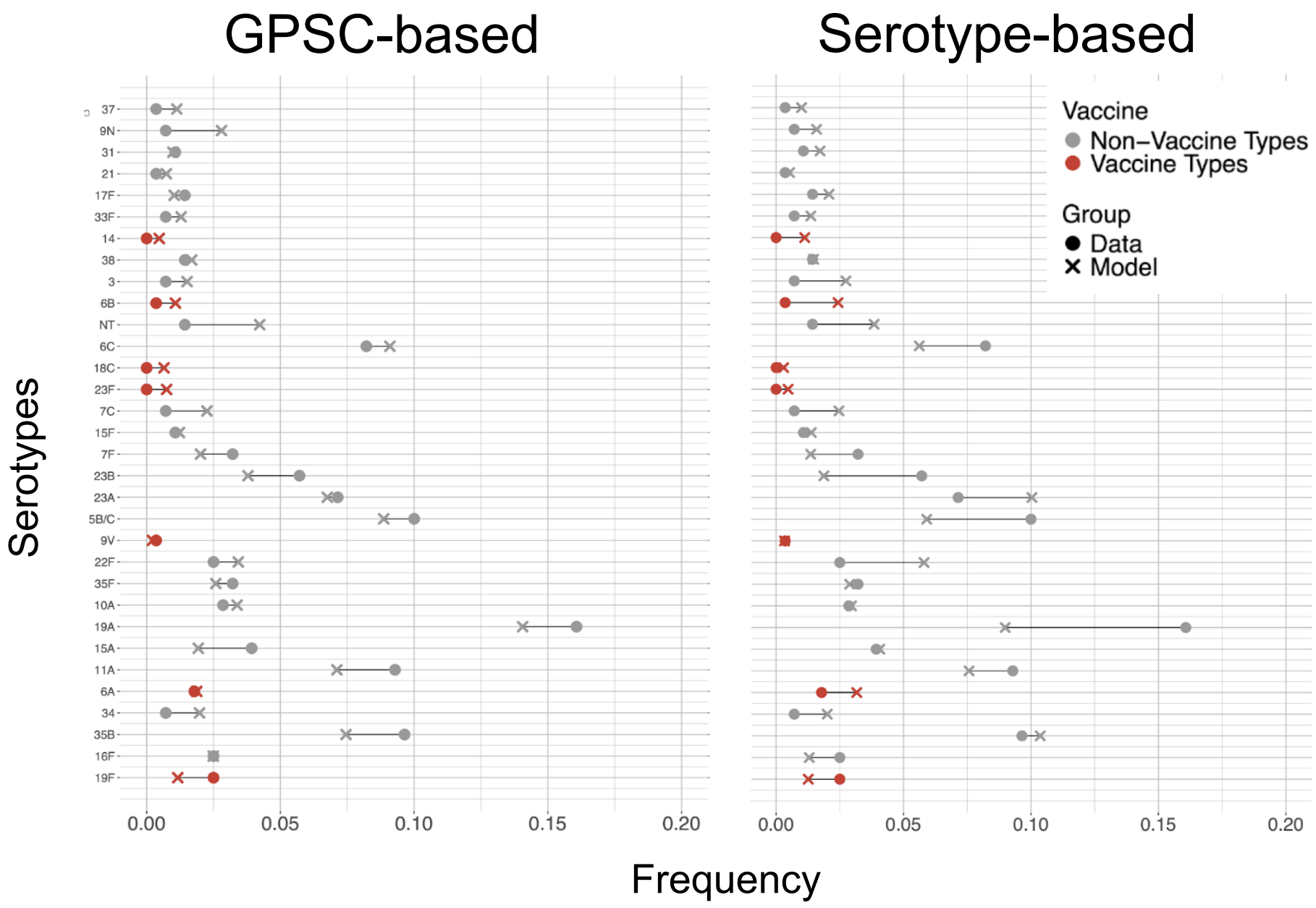

Fig. S9: Comparison of GPSC-based model and serotype-based model.

Table S2: Functional annotation of genes that were found to be under NFDS in Massachusetts, Southampton, and Kathmandu. 14 genes were found to be under NFDS in all countries, out of which 8 had a functional annotation.

Functional annotation
phosphoenolpyruvate–glycerone phosphotransferase
DUF3567 domain-containing protein
RelE/StbE replicon stabilization toxin
two-peptide bacteriocin subunit BlpN
Bacteriocin
AzlD domain-containing protein
Gp24
Type I restriction-modification system, S subunit

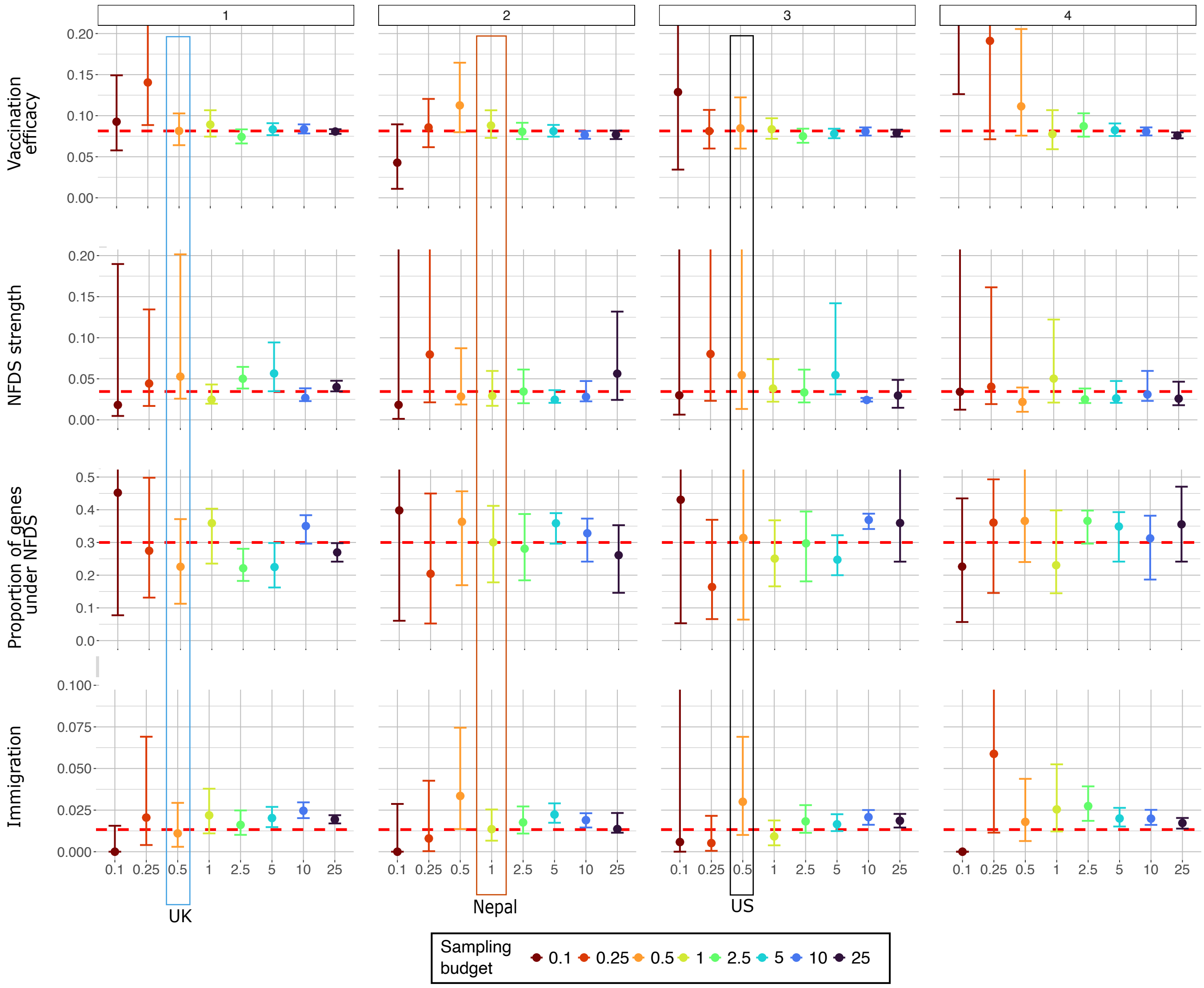

Fig. S10: Parameter estimates and credible intervals for fits on synthetic data with different sampling budgets and frequencies. The x-axis and colours show simulated sampling budgets equivalent to sampling 0.1% to 25% annually of the model population of 15,000. Y-axis shows parameter values for the four different parameters. Panels show sampling every one, two, three or four years. Original parameter values used to produce synthetic data are shown as red dashed line. Sampling strategies closest to the ones in Southampton (UK), Massachusetts (US), and Kathmandu (Nepal) are highlighted.
